# A pH-responsive bi-MIL-88B MOF coated with folic acid-conjugated chitosan as a promising nanocarrier for targeted drug delivery of 5-Fluorouracil

**DOI:** 10.3389/fphar.2023.1265440

**Published:** 2023-09-06

**Authors:** Muhammad Usman Akbar, Saadullah Khattak, Malik Ihsanullah Khan, Umair Ali Khan Saddozai, Nemat Ali, Abdullah F. AlAsmari, Muhammad Zaheer, Muhammad Badar

**Affiliations:** ^1^ Gomal Center of Biochemistry and Biotechnology, Gomal University, Dera Ismail Khan, Pakistan; ^2^ Henan International Joint Laboratory of Nuclear Protein Regulation, School of Basic Medical Sciences, Henan University, Kaifeng, China; ^3^ Institute of Molecular Biology and Biotechnology, The University of Lahore, Lahore, Pakistan; ^4^ Department of Preventive Medicine, Institute of Bioinformatics, Henan Provincial Engineering Center for Tumor Molecular Medicine, School of Basic Medical Sciences, Henan University, Kaifeng, China; ^5^ Department of Pharmacology and Toxicology, College of Pharmacy, King Saud University, Riyadh, Saudi Arabia; ^6^ Department of Chemistry and Chemical Engineering, Syed Babar Ali School of Science and Engineering, Lahore University of Management Sciences (LUMS), Lahore, Pakistan

**Keywords:** metal-organic framework, folic acid -chitosan, stimuli responsive, drug delivery, targeted therapy, anticancer

## Abstract

Cancer has remained one of the leading causes of death worldwide, with a lack of effective treatment. The intrinsic shortcomings of conventional therapeutics regarding tumor specificity and non-specific toxicity prompt us to look for alternative therapeutics to mitigate these limitations. In this regard, we developed multifunctional bimetallic (FeCo) bi-MIL-88B-FC MOFs modified with folic acid—conjugated chitosan (FC) as drug delivery systems (DDS) for targeted delivery of 5-Fluorouracil (5-FU). The bi-MIL-88B nanocarriers were characterized through various techniques, including powder X-ray diffraction, scanning electron microscopy, energy-dispersive X-ray, thermogravimetric analysis, and Fourier transform infrared spectroscopy. Interestingly, 5-FU@bi-MIL-88B-FC showed slower release of 5-FU due to a gated effect phenomenon endowed by FC surface coating compared to un-modified 5-FU@bi-MIL-88B. The pH-responsive drug release was observed, with 58% of the loaded 5-FU released in cancer cells mimicking pH (5.2) compared to only 24.9% released under physiological pH (5.4). The *in vitro* cytotoxicity and cellular internalization experiments revealed the superiority of 5-FU@bi-MIL-88B-FC as a highly potent targeted DDS against folate receptor (FR) positive SW480 cancer cells. Moreover, due to the presence of Fe and Co in the structure, bi-MIL-88B exhibited peroxidase-like activity for chemodynamic therapy. Based on the results, 5-FU@bi-MIL-88B-FC could serve as promising candidate for smart DDS by sustained drug release and selective targeting.

## 1 Introduction

The emergence of nanomedicine as a next-generation technology has brought a revolution in battling diseases, particularly cancer (van der Meel et al., 2019). Cancer, for decades, has remained the leading cause of death worldwide after cardiovascular disease (Dibden et al., 2020). Despite advances in early diagnosis and associated treatments, current anticancer therapies rely heavily on invasive surgical procedures, radiotherapy, and chemotherapy (Das et al., 2020). These procedures are endemic to the problems of unwanted toxicity, insufficient drug delivery, premature drug degradation, and cancer recurrence due to incomplete eradication (Ulldemolins et al., 2021). The field of nanomedicine tries to mitigate such limitations by using smart nanodevices to transport therapeutic molecules specifically to cancer cells reducing off-target side effects (Akbarzadeh et al., 2021; Manzari et al., 2021; Surekha et al., 2021; Bäumer et al., 2022). Drug delivery systems based on nanodevices have become increasingly popular due to their advantages of improved drug loading, stable transfer of drugs to the target site, and reduced dosage requirement (Li Y. et al., 2022; Wang et al., 2022a; Yu et al., 2022; Rana et al., 2023). In this regard, various nanomaterials have been explored as drug delivery systems comprising carbon nanotubes (CNTs), liposomes, hydrogels, Layered double hydroxides (LDHs), dendrimers and metal-organic frameworks (MOFs) (Hesse et al., 2013; Chen et al., 2020; Zhang et al., 2020; Anisimov et al., 2022; Yang et al., 2022; Zhao et al., 2023). Among others, MOFs, due to their exceptional characteristics, have recently gained much attention for their potential in gas sorption, catalysis, sensing, and drug delivery (Okur et al., 2021; Abbas et al., 2023; Oh et al., 2023; Ye et al., 2023). MOFs are crystalline materials formed by self-assembling inorganic (metal ion/clusters) and organic linkers through coordination chemistry (Vodyashkin et al., 2023). They are highly diverse structures with tunable surface chemistry, adjustable pores, and high surface areas reaching up to 10,000 m^2^/g (Adegoke and Maxakato, 2021; Altintas et al., 2022; Yin et al., 2022). Moreover, through pre or post-synthetic modifications, MOFs can respond to various stimuli (pH, temperature, redox reaction, and ATP) (Wang et al., 2020; Sun and Davis, 2021). Upon encountering such stimuli, MOFs undergo structural alterations allowing them to release their encapsulated drug molecules (Zhao et al., 2021). Various MOFs such as MIL-101 (MIL = Material Institute Lavoisier), UiO-66 (UiO = University of Oslo), and ZIF-8 (ZIF = Zeolite Imidazolate Framework) have been successfully deployed in the past as stimuli-responsive smart DDS for chemotherapy (Abánades Lázaro et al., 2020; Karimi Alavijeh and Akhbari, 2020; Yan et al., 2020). The metal nodes in MOFs also act as catalytic centers performing peroxidase-like (POD) reactions to induce reactive oxygen species (ROS) mediated stress in cancer cells for chemodynamic therapy (Di et al., 2023). In this regard, mixed-metal MOFs have shown higher POD performance than mono-metallic MOFs due to the excellent M^III^/M^II^ cycling frequency and efficient electron transfer capability (Lyu et al., 2017; Wen et al., 2021). The performance of MOFs for drug delivery applications could also be improved by making MOFs-composites through surface modification or encapsulating MOFs in biodegradable materials (e.g., Biopolymers) (Ge et al., 2022).

Compared to other polymers used for biodegradable coatings in targeted DDS, chitosan (CS) has recently attracted much attention due to its cationic character, biodegradable nature, pH sensitivity, efflux pump inhibition, and higher cellular permeability (Aibani et al., 2021; Sathiyaseelan et al., 2021). The repeated amine groups found in the structure of CS are responsive towards tumor microenvironment mimicking acidic media and cause swelling of the system to release loaded cargo (Lv et al., 2016; Chen et al., 2017). The tumor specificity of the DDS modified with CS could further be improved by functionalizing it with active targeting ligands like folic acid (FA) (Nemati et al., 2021). Since FA receptors are exclusively overexpressed in most tumor cells, CS functionalization with FA could help DDS internalize into the cells through receptor-mediated endocytosis (İnce et al., 2020). However, the application of CS-based DDS as stand-alone nanocarriers is limited due to their rapid degradation and higher swelling degree leading towards pre-mature drug release (Peers et al., 2020). Thus, making a composite of CS with other materials is termed beneficial to improve the system’s overall efficiency (El Leithy et al., 2019).

5-Fluorouracil (5-FU) is a pyrimidine analog anticancer drug that exerts its cytotoxic effects through DNA/RNA incorporation, causing apoptosis in cancer cells (Guo et al., 2020). However, it has a rapid degradation rate (5–10 min) which hampers its broad clinical efficacy (Longley et al., 2003). The non-specific nature of the 5-FU and lack of suitable carriers further aggravate the situation by causing side effects such as diarrhea, cardiac toxicity, mucositis, dermatitis, and myelosuppression (Chang et al., 2012). Therefore, encapsulation of 5-FU in suitable carriers to avoid unnecessary side effects has been in focus (Valencia-Lazcano et al., 2023). For this, FeCo based bi-MIL-88B nanocarriers were synthesized in the current study due to their flexible structure, high surface area, and biocompatible nature of the components (Horcajada et al., 2012). The bi-MIL-88B nanocarriers exhibited a higher 5-FU loading capacity of 29.8wt%. After loading, these nanocarriers were coated with FA-conjugated CS (FC) to endow them with an extra-gated obstruction in premature drug release and folate receptor-associated cellular uptake. The pH-responsive 5-FU release was realized against the tumor-mimicking environment (pH = 5.2) and a normal physiological environment (pH = 7.4). The *in vitro* cytotoxicity and cellular uptake of the FC-coated bi-MIL-88B were checked against HEK-293 (FR negative) and SW480 (FR positive) cell lines. Moreover, the Fe^III^ and Co^II^ based trinuclear clusters in MIL-88B act as catalytic centers for *in situ* peroxidase-like activity.

## 2 Materials and methods

All the chemicals used in the study were of analytical grade and used as received. Iron (III) nitrate nonahydrate (Fe(NO_3_)_3_·9H_2_O), Cobalt (II) nitrate hexahydrate (Co(NO_3_)_3_·6H_2_O), Sodium acetate trihydrate (CH_3_COONa·3H_2_O), Terephthalic acid, 5-Fluorouracil (5-FU), N, N-dimethylformamide (DMF), 3,3′,5,5′-Tetramethylbenzidine (TMB), Phosphate buffer saline (PBS) tablets, Chitosan (CS), Folic Acid (FA), 1,1′-Dioctadecyl-3,3,3′,3′tetramethyl indocarbocyanine perchlorate (Dil), N-hydroxysuccinimide (NHS), 1-ethyl-3-(3-dimethyl aminopropyl) carbodiimide (EDC), dimethyl sulfoxide (DMSO), and Glacial acetic acid used were manufactured of Sigma-Aldrich. Roswell park memorial institute (RMPI-1640) medium, 3-(4,5-Dimethylthiazol-2-yl)-2,5-Diphenyltetrazolium Bromide (MTT), L-glutamine, Penicillin-Streptomycin (pen-strep), Alexa fluor™ 488 Phalloidin and Fetal bovine serum (FBS), were manufactured of Gibco, Invitrogen.

### 2.1 Characterization

A powder X-ray diffraction (PXRD) pattern was obtained to perform crystal structure analysis using BRUKER (D2 Phaser) with Ni-filtered Cu-Kα irradiation (λ = 1.5406 Å) over 2θ range from 5° to 50°. FEI NOVA Nano 450 scanning electron microscope (SEM) equipped with an energy dispersive X-ray spectroscope (EDX) was used to analyze the morphology of the samples. The samples’ Zeta potential (ZP) was obtained through Zetasizer (Nano ZS, Malvern) at room temperature in water. N_2_ adsorption-desorption isotherm was obtained to Brunauer-Emmett-Teller (BET) surface area and porous makeup of the samples using Quantachrome Nova 2200e. Infrared studies were performed using Bruker Alpha Platinum ATR between the 500–4,500 cm^-1^ range. Thermogravimetric analysis (TGA) was obtained through the TA instrument under an N_2_ atmosphere in a temperature ranging from 10°C to 600°C with a heat ramp of 10°/min. UV-Vis spectrophotometry was used to characterize drug loading/release and TMB oxidation studies by Shimadzu UV-1800 spectrophotometer. The cellular uptake fluorescence studies were performed through confocal laser scanning microscope (CLSM) model ZEISS LSM—880, Jena, Germany.

### 2.2 Synthesis of bi-metallic cluster

The synthesis of bi-metallic acetate cluster FeCo(*μ*_3-_O) was performed using a previously reported method with slight modifications (Sanchez-Lievanos et al., 2020). Briefly, 0.022 mol (3 g) of CH_3_COONa.3H_2_O were dissolved in 5 mL of deionized water and was called solution-A. On the other hand, a solution-B of Fe and Co was prepared by dissolving 0.0014 mol (0.571 g) of Fe(NO_3_)_3_.9H_2_O and 0.007 mol (2.07 g) of Co(NO_3_)_3_.6H_2_O in 5 mL of deionized water and was kept on stirring after filtration. Later, solution-A was added dropwise into the thoroughly stirred solution-B, and the mother mix was kept on stirring for 24 h at room temperature. After 24 h of stirring, the light brown precipitates were collected through filtration and washed thrice with small amounts of water and ethanol. After washing, the collected product was kept to air dry at room temperature.

### 2.3 Synthesis of bi-MIL-88B

To synthesize bi-MIL-88B MOFs from pre-synthesized FeCo-*μ*_3_O clusters, equimass of Terephthalic acid (100 mg) and FeCo-*μ*_3_O (100 mg) were separately dissolved in vials containing 9 mL of DMF each through sonication. After dissolution, the terephthalic acid solution was added into the FeCo-*μ*_3_O containing solution under stirring. An additional 1 mL of the glacial acetic acid as a modulating agent was added to the mother solution. The whole mixture was homogenously dissolved and inserted into a Teflon-lined autoclave for incubation at 120°C for 24 h. After 24 h of reaction, bi-MIL-88B MOF precipitates were isolated through centrifugation and later washed thrice with DMF and distilled ethanol to remove any unreacted linker present in the structure.

### 2.4 Preparation of folic acid-conjugated chitosan (FC)

The folic acid conjugated chitosan (FC) was synthesized using a previously reported method (Hu et al., 2017). In this method, amine groups of CS were conjugated to the FA by NHS-EDC chemistry. Briefly, a solution of FA (0.16 mmol, 7, 150 mg) was prepared through dissolution in 40 mL of anhydrous DMSO at room temperature. After that, NHS (3.36 mmol, 380 mg) and EDC (3.36 mmol, 645 mg) were added to the solution and stirred for 2 hours at room temperature. The solution turned into red brown colored ester solution of DMSO containing activated FA. In the second step, a solution of CS was prepared by dissolving 60 mg of CS in 15 mL of sodium acetate buffer (pH = 7.4) containing 0.1 M acetic acid. Later, the activated FA solution of DMSO was added dropwise into the CS solution at room temperature under dark conditions. The solution was allowed to stir for 24 h. After this time, the pH of the solution was adjusted to 9.0 through the slow addition of 0.1 M sodium hydroxide. In the end, the obtained mixed solution was dialyzed in PBS for 3 days to remove phosphoric acid salt, and finally, FC conjugates were obtained through freeze drying.

### 2.5 Drug loading

Before drug loading, bi-MIL-88B nanocarriers were activated under vacuum for 24 h at 100°C to eliminate some of the coordinated solvent molecules occupying the pores. Briefly, 100 mg of bi-MIL-88B were dispersed into a 30 mL concentrated 5-FU (6,000 ppm) solution in ethanol. The solution was put on an orbital shaker at room temperature for 48 h. After that, the drug-loaded 5-FU@bi-MIL-88B MOFs were isolated through centrifugation and the supernatant was analyzed for the remaining drug. The drug loading capacity (DLC) and drug loading efficiency (DLE) of the nanocarriers were determined using a calibration curve of 5-FU in ethanol (λ_max_ = 265 nm) (Supplementary Figure S8) according to the following formulas (Parsaei and Akhbari, 2022a);

$$DLC\;\left(wt\%\right)=\frac{weight\;of\;loaded\;drug}{weight\;of\;drug\;loaded\;MOFs}\times 100$$ (1)

$$DLE\;\left(wt\%\right)=\frac{weight\;of\;loaded\;drug}{total\;weight\;of\;feeded\;drug}\times 100$$ (2)

### 2.6 Fabrication of 5-FU@bi-MIL-88B-FC

To prepare the final composite, FC (20 mg) was dissolved in 4 mL of an acetic acid solution (pH 6.0) under stirring for 24 h to form a homogenous solution. After that, the homogenous FC solution was added to the saturated ethanolic solution of 5-FU (20 mL) containing 100 mg 5-FU@bi-MIL-88B dispersed nanocarriers. Finally, the master mix was stirred at room temperature for 24 h. Later, the resultant FC-coated drug carriers were collected through centrifugation and rinsed twice with ethanol and ultrapure water. After rinsing, the final products were allowed to dry at room temperature under a vacuum for 24 h.

### 2.7 Drug release

The pH-responsive drug release from samples was realized against TME (pH 5.2) and physiological environment (pH 7.4) mimicking PBS solutions. Briefly, 60 mg of 5-FU@bi-MIL-88B and 5-FU@bi-MIL-88B-FC were dispersed in a dialysis bag (3.5 kDa MWCO) containing a small amount of PBS. Later, the dialysis bag containing drug-loaded nanocarriers was placed in a beaker containing 60 mL of PBS (pH 5.2 and 7.4). The drug release was performed through dialysis at 37°C under mild stirring. At predetermined intervals, 1 mL of the dialysate solution was pipetted out and replaced with the same amount of fresh PBS to maintain the total volume constant. The withdrawn samples were analyzed through a UV-Vis spectrophotometer, and the concentration of the released drug was determined according to the calibration curve of 5-FU in PBS (Supplementary Figure S9). The experiments were performed in duplicate and the final results were plotted through averaging. The following equations were used to obtain the cumulative 5-FU release percentage:

$$Drug\;release\;\left(cumulative\;\%\right)=\frac{Rt}{Rf}\times 100$$

Where Rt denotes the 5-FU concentration released at time t and Rf represents the total amount of 5-FU loaded on the nanocarriers.

### 2.8 Cell culture

Human embryonic kidney cells (HEK-293 cells) and human colon cancer (SW480 cells) were obtained from The University of Lahore (UOL) Cell Culture Collection (UCCC). The cells were cultured in RMPI-1640 media supplemented with 1% Pen-strep (100 IU/ml penicillin and 100 μg/mL streptomycin), 10% Hi-FBS, and 2 mM L-glutamine in a humidified incubator with 5% CO_2_ at 37°C.

### 2.9 Cell cytotoxicity assay

The *in vitro* cytotoxicity of the 5-FU, bi-MIL-88B, 5-FU@bi-MIL-88B and 5-FU@bi-MIL-88B-CS was evaluated by the MTT assay. Briefly, the HEK293 and SW480 cells were seeded in a 96-well plate at a 1 × 10^4^ density and incubated for 24 h in a CO_2_ incubator at 37°C. After 24 h, the cell culture medium was removed and different concentrations of the test samples (7.81—500 μg/mL) dissolved in the culture medium supplied to the cells and allowed for 48 h of incubation. After incubation, 10 μL of MTT (12 mM) reagent was further supplied to each well and the cells were further incubated for another 4 h. Later, the medium was removed and DMSO (100 μL) was added to each well. The absorbance was recorded by PerkinElmer Enspire 2300 multimode reader at 570 nm. The experiments were conducted in triplicated and the final results were presented through averaging. The IC_50_ values were calculated by a non-linear regression model using GraphPad Prism 8 (San Diego, United States).

### 2.10 Cellular uptake studies

Confocal laser scanning microscopy was used to study the cellular uptake of nanocarriers. For CLSM imaging, SW480 cells at a density of 3 × 10^4^ were seeded and grown on a glass coverslip in a 24-well plate for 24 h. After incubation for a predetermined time, the original medium was replaced with fresh medium containing Dil@bi-MIL-88B and Dil@bi-MIL-88B-CS (80 μg/mL) and incubated for an additional 12 h. Dil was used as a fluorescent probe to detect the internalization of the nanocarriers. Later, cells were washed twice with PBS and fixed through 4% formalin. The DAPI and Alexa flour 488 phalloidine were used to stain the nuclei and cytoskeleton of the cells. Finally, the cells were visualized under CLSM.

### 2.11 Peroxidase-like activity

The peroxidase-like property of synthesized nanocarriers was studied through the TMB oxidation methodology. Briefly, 5 mL of PBS (pH 5.2) was prepared by adding different amounts of bi-MIL-88B (0, 10, 20, 40, 60, and 80 μg/mL), H_2_O_2_ (1 mM) and TMB (0.25 mM) and allowed to incubate for 10 min at 37 °C. After that, samples were analyzed through a UV-Vis spectrophotometer at 652 nm wavelength related to the oxidized form of the TMB. Moreover, mechanistic studies on the performance of bi-MIL-88B nanocarriers were performed by varying the temperature (30°C—60°C) and pH (4—8) of the solution with concentrations of TMB (0.25 mM), H_2_O_2_ (1 mM) and bi-MIL-88B (50 μg/mL) kept constant.

### 2.12 Statistical analysis

The statistical analysis carried out in the study was performed through GraphPad Prism 8.0. The MTT data were shown as mean ± standard deviation. The statistically significant values of different groups were obtained through the Kruskal-Wallis test, followed by Dunn’s multiple comparison analysis. The degree of significance of the treated groups against the control is represented as ****p ≤ 0.0001, ***p ≤ 0.001, **p ≤ 0.01, and *p ≤ 0.05.

## 3 Results and discussions

### 3.1 Synthesis and characterization of bi-MIL-88B

**SCHEME 1:**
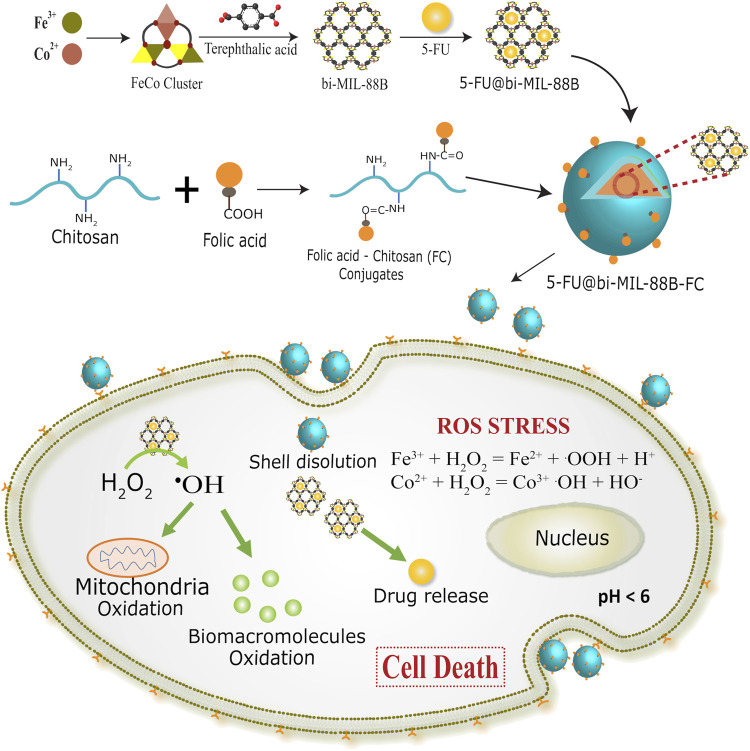
Synthetic scheme of 5-FU@bi-MIL-88B and folate receptor (FR) mediated endocytosis in SW480 (FR positive) cells.

The bi-metallic (FeCo) bi-MIL-88B MOFs were synthesized using a previously published two-step secondary building unit (SBU) approach (Iqbal et al., 2021). A FeCo-*μ*_3_O trinuclear cluster with metal ions connected to central oxygen (*μ*_3-_O) in a trinuclear fashion was synthesized in the first step (Akbar et al., 2022). These metal ions are stabilized through the coordinated acetate ligands and solvent molecules at their terminal positions. In the second step, FeCo-*μ*_3_O cluster is reacted with the terephthalic acid as the organic ligand. During the reaction, the terephthalic acid attaches to the metal ions by replacing the acetate ligands in a dissociative manner to form a bi-MIL-88B MOFs (Liu et al., 2016). Compared to SBU route, mixed-metal MOF synthesis through conventional one-pot synthesis or postsynthetic modifications (PSMs) method is tricky and results in mixed phase MOFs with unwanted altered physico-chemical properties (Li et al., 2013). Moreover, these methodologies provide less control over the reproducibility of the same MOFs and often generate unwanted metal oxides or even amorphous structures (Wongsakulphasatch et al., 2015). While, the SBU route exhibits certain advantages over others as the concentration of metals in the final MOFs can be precisely controlled avoiding the generation of unwanted metal oxides. Moreover, stable incorporation of the pre-synthesized mixed-metal SBU into the final MOF allows excellent reproducibility with predictable incorporation of the second metal (Co) with stoichiometric ratio of Fe and Co (2 : 1) (Peng et al., 2017). The synthetic approach of bi-MIL-88B, drug loading, FC coating, and mechanism of action are illustrated in Scheme 1.

**FIGURE 1:**
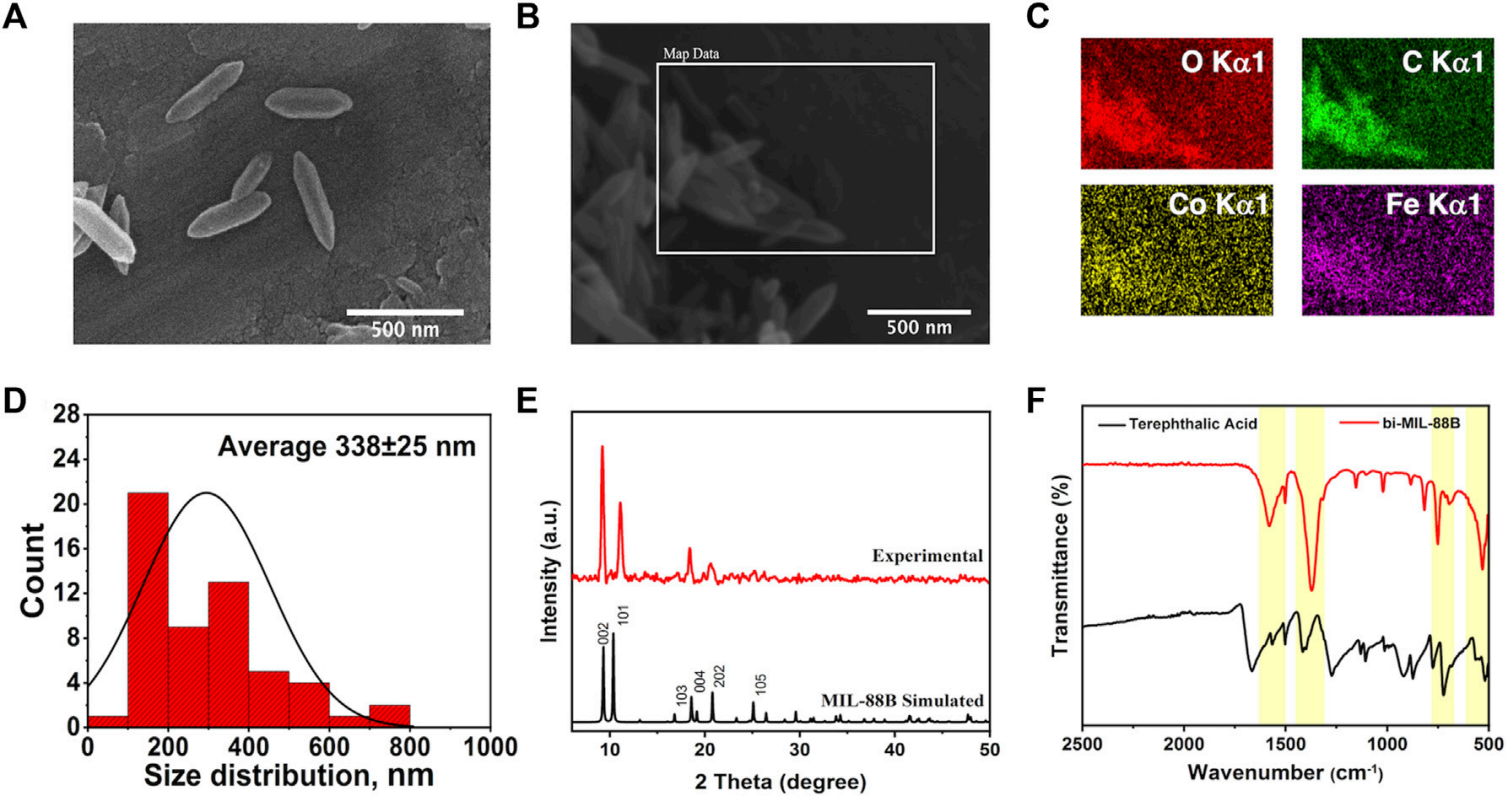
**(A–B)** SEM images; **(C)** Elemental Maps; **(D)** Size distribution chart of bi-MIL-88B nanocarriers; **(E)** PXRD pattern of simulated and experiment bi-MIL-88B; **(F)** FT-IR spectra of terephthalic acid and bi-MIL-88B.

The SEM analysis was performed to observe the morphological features of the synthesized samples. As seen in Supplementary Figures S1A, B, the FeCo clusters exhibited a jumble of rocks type appearance having undefined morphology. However, upon reaction with the organic linker, the resulting bi-MIL-88B MOFs revealed hexagonal rod-like morphology resembling the pure MIL-88B MOFs reported in the literature (Figures 1A, B) (Cai et al., 2016). The average size of the bi-MIL-88B was around 338 ± 30 nm, evaluated through average aspect ratio and size distribution analysis by DLS method (Figure 1D; Supplementary Figure S5). The EDX, elemental map and ICP-OES analysis was performed to analyze the elemental composition of the FeCo-cluster and bi-MIL-88B. The EDX spectra and elemental maps showed the homogenous distribution of Fe and Co ions having a stoichiometric ratio of Fe to Co (2 : 1) in the synthesized cluster (Supplementary Figures S1D, S2A) and bi-MIL-88B (Figure 1C; Supplementary Figure S2B).

Moreover, the ICP-OES analysis also revealed the homogenous distribution of Fe and Co in both samples with a ratio of 2:1 (Supplementary Figure S1). The presence of a similar stoichiometric ratio of both metals in the bi-MIL-88B indicates that the FeCo clusters retained their structural traits in the final product without any deformities. FT-IR analysis was performed to evaluate the major linkages in the samples. In the case of monometallic Fe-based MIL-88B, the Fe_3_O trinuclear cluster in the structure exhibits metal-oxygen bond vibrations around 600 cm^-1^. However, when one Co is incorporated into the cluster, the D_3h_ symmetry of Fe_3_O breaks into C_2v_, evident by the emergence of two new vibrational stretching around 734 cm^-1^ and 528 cm^-1^ related to FeCo-O bonds in the cluster (Supplementary Figure S3) (Iqbal et al., 2019). The vibrational bands found around 1,590 cm and 1,420 cm^-1^ in the cluster are related to the carboxyl groups of the coordinated acetate ligands (Zhang et al., 2012). The vibrational stretching in bi-MIL-88B MOFs found at 1,592 cm^-1^ and 1,386 cm^-1^ were related to -COO stretching of the coordinated linker (Liu et al., 2013).

The PXRD analysis revealed the crystal structure and phase purity of the samples. The characteristic peaks of the synthesized FeCo cluster’s PXRD pattern matched well with the simulated one (Supplementary Figure S1C) (Sanchez-Lievanos et al., 2020). The bi-MIL-88B exhibited highly crystalline phase purity with distinctive peaks at 9.3°, 10.2°, and 11.6° related to 002, 100, and 101 planes also found in the simulated MIL-88B MOF (Figure 1F) (Horcajada et al., 2008). The porous makeup of the bi-MIL-88B nanocarriers was studied by N_2_ adsorption-desorption analysis at 77K (Supplementary Figure S4A). The BET-specific surface area of the bi-MIL-88B was 86 m^2^/g with an average pore diameter and volume of 1.9 nm and 0.21 cc/g (Supplementary Figure S4B). The lower surface area of the nanocarriers could be due to shrinkage of the structure upon solvent removal during thermal activation (Ma et al., 2013). The bi-MIL-88B MOFs have a flexible structure and tend to shrink/expand on the removal/addition of the guest molecules, known as a reversible breathing effect (Cao et al., 2022).

### 3.2 Fabrication of 5-FU@bi-MIL-88B-FC

**FIGURE 2:**
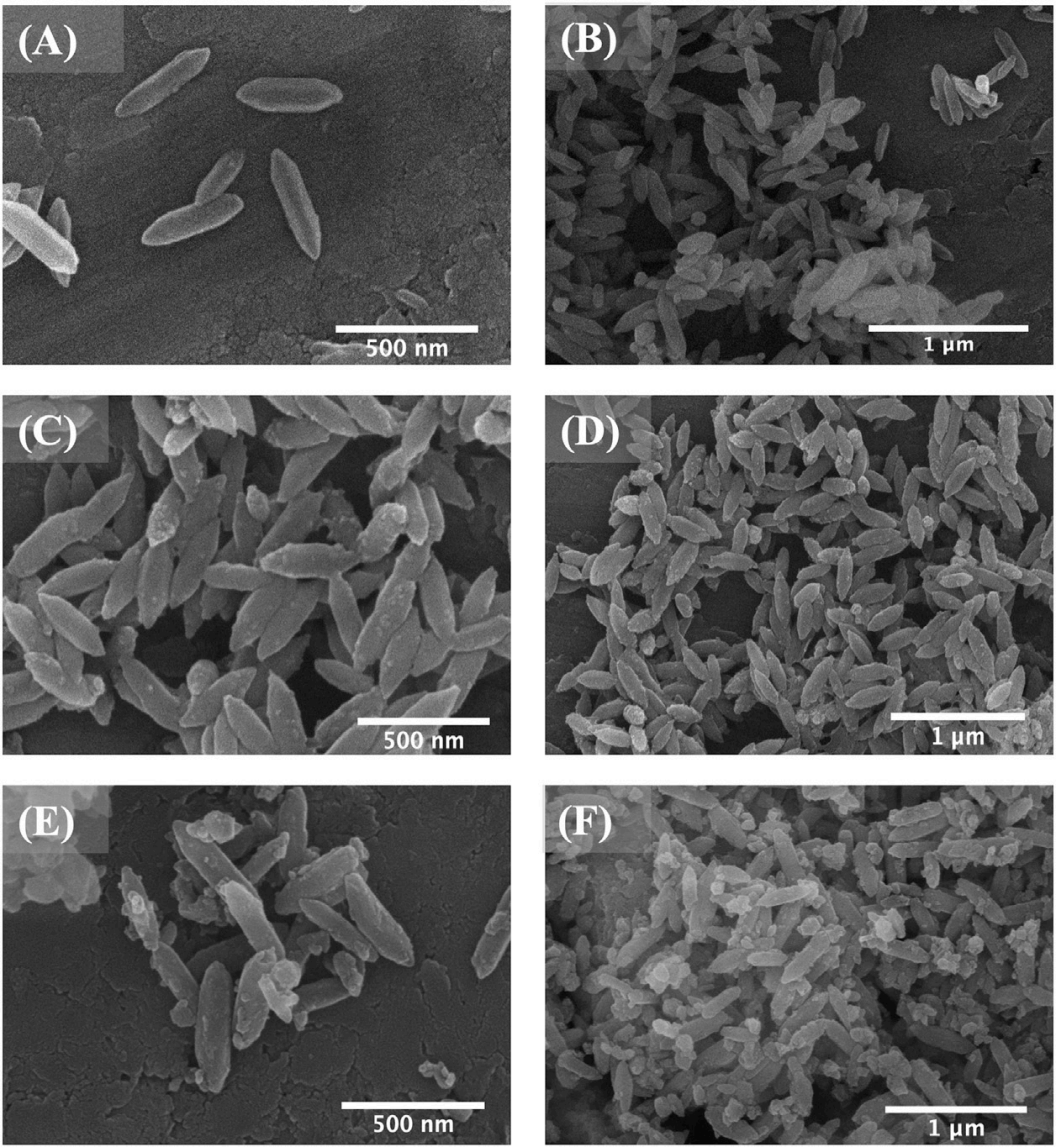
SEM images of bi-MIL-88B **(A, B)**; 5-FU@bi-MIL-88B **(C, D)**; and 5-FU@bi-MIL-88B-FC **(E, F)**.

SEM analysis was used to study the morphological changes after the drug impregnation and subsequent FC coating. As shown in Figure 2, bi-MIL-88B, 5-FU@bi-MIL-88B, and 5-FU@bi-MIL-88B-FC, the drug-loaded and FC-coated nanocarriers exhibited similar morphology to the unmodified MOFs. However, 5-FU@bi-MIL-88B reflects some swelling crystals due to the drug impregnation and reversible expansion. After FC coating, 5-FU@bi-MIL-88B-FC showed less aggregation than 5-FU@bi-MIL-88B, which aggregated upon drug impregnation (Figure 2).

**FIGURE 3:**
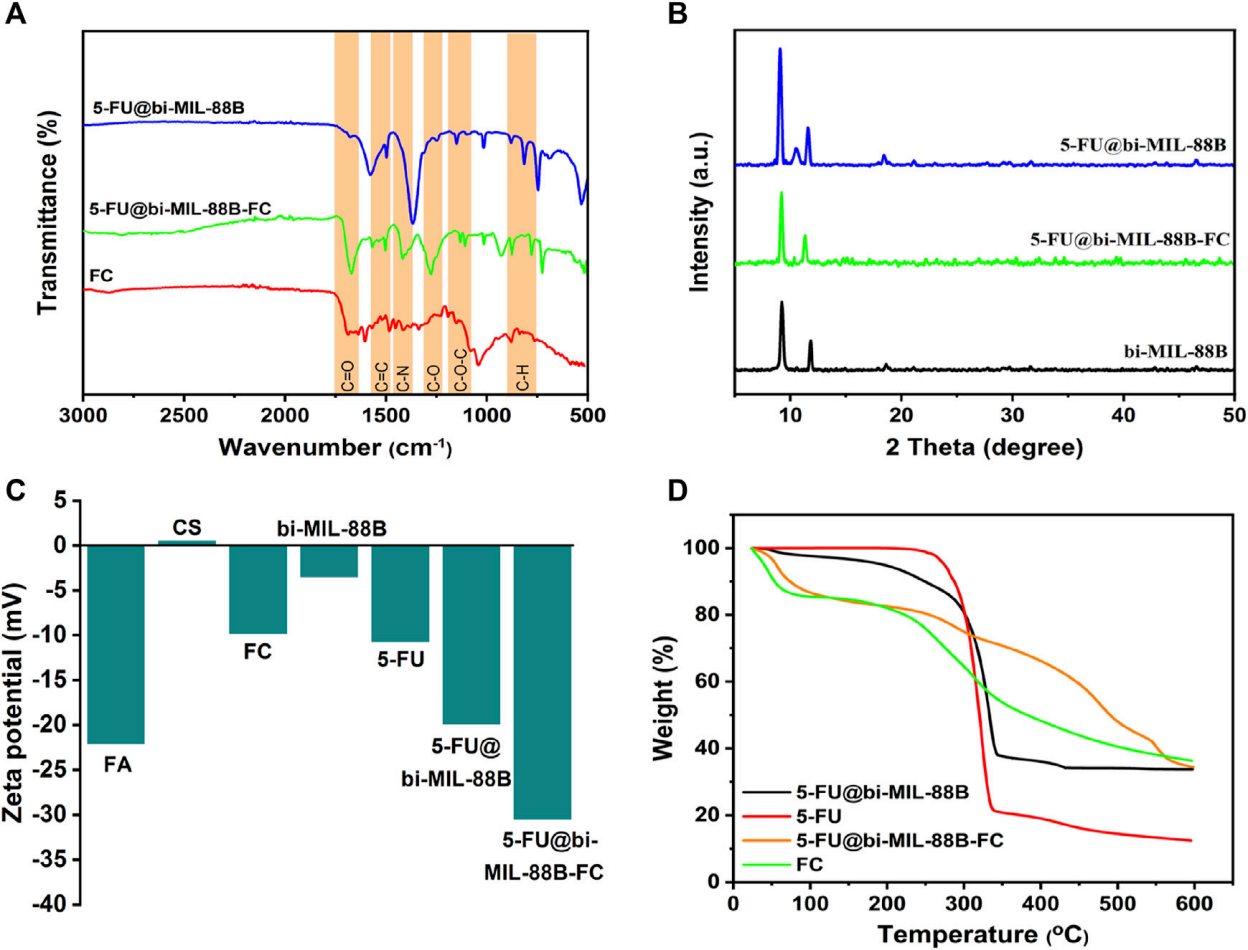
**(A)** FT-IR spectra; **(B)** PXRD patterns; **(C)** Zeta potential values; and **(D)** TGA patterns of the samples.

The FT-IR studies further confirmed the incorporation of the 5-FU and the final synthesis of the FC-coated composite. As seen in the FT-IR spectra of 5-FU (Supplementary Figure S6), the vibrational peaks around 1,245 cm^-1^ and 1740 cm^-1^ are related to the C—N and C—O stretching, and the peaks between 800 cm^-1^ to 540 cm^-1^ represent C—F deformations (Chowdhuri et al., 2016). The characteristic peaks of 5-FU, when compared with bi-MIL-88B, can also be seen in the drug-loaded 5-FU@bi-MIL-88B samples confirming the successful drug incorporation. After the drug encapsulation, the second step involved the synthesis of folic acid–conjugated chitosan (FC) and subsequent composite 5-FU@bi-MIL-88B-FC. The synthesis of FC can be verified by comparing the FT-IR spectra of CS, FA and final conjugated FC. As seen in Supplementary Figure S7, in the FT-IR spectrum of CS, the peaks at 3,360 cm^-1^, 2922 cm^-1^ and 2875 cm^-1^ are attributed to the N—H and asymmetric/symmetric vibrations of C—H groups. The peaks around 1,060 cm^-1^ represent C—O stretching, and vibrational bands around 1,322 cm^-1^ and 1,650 cm^-1^ are related to C—N and C=O bonds in the CS. While the peak at 1,154 cm^-1^ represents asymmetric stretching modes of C—O—C in the CS spectrum (Chen et al., 2011; Al-Nemrawi et al., 2022). Whereas the characteristic peaks at 1,695 cm^-1^, 1,480 cm^-1^, 1,230 cm^-1^ and 1,170 cm^-1^ in the FT-IR spectra of FA are attributed to C=O, C=C, C—O, and C-N vibrational stretching. The 830 cm^-1^ and 750 cm^-1^ bands represent aromatic rings’ out-of-plane C—H bond stretching (Parsaei and Akhbari, 2022b). Most of the characteristic peaks of CS and FA are observed in the FT-IR spectra of FC, which confirms the conjugation of FA to the CS in the final product (Supplementary Figure S7) (Chanphai et al., 2017). Moreover, the characteristic peaks of FC conjugates are also visible in the 5-FU@bi-MIL-88B composite confirming the successful coating of the 5-FU@bi-MIL-88B nanocarriers with the FC (Figure 3A). The influence of the 5-FU encapsulation and FC coating on the structural properties of the bi-MIL-88B was observed through the PXRD analysis. According to Figure 3B, no significant alteration in the PXRD patterns of the 5-FU@bi-MIL-88B and 5-FU@bi-MIL-88B-FC was observed compared to the pure bi-MIL-88B. A minor decrease in the diffraction angle of the peak related to the 101 plane from 11.6 to 11.2 can be attributed to the pore expansion by 5-FU loading due to the reversible breathing effect (Horcajada et al., 2011). The reduction in the overall peak intensities of the 5-FU@bi-MIL-88B-FC nanocarriers could be due to the external coating by the FC (Shi et al., 2018).

The ZP of the nanocarriers plays an essential role in deciding the stability and adhesion to the cells (Ishihara et al., 2015). The ZPs of the CS, FA, FC, 5-FU, bi-MIL-88B, 5-FU@bi-MIL-88B, and 5-FU@bi-MIL-88B were 0.47, −22.1, −9.83, −10.7, −3.52, −19.9 and −30.5 respectively (Figure 3C). The positive ZP of CS is due to the cationic amino groups, and the negative ZP of the FA can be ascribed to the anionic carboxyl groups of FA (Song et al., 2013). The shift to the higher negative ZP value after the FC coating of 5-FU@bi-MIL-88B can be related to the anionic properties of the FC conjugates. The higher ZP values for nanocarriers are beneficial as the highly charged particles tend to repulse each other limiting agglomeration. A lower ZP value results in coagulation due to the weaker repulsion force being overtaken by the attraction force between the charged particles. Moreover, nanoparticles are found in stabilized dispersions with an optimal ZP value of −30 mV (Samimi et al., 2019). The surface charge of the nanocarriers also plays a significant role in the cellular uptake of the nanocarriers. The nanocarriers with cationic character are usually internalized into the cell via caveolae-mediated endocytosis and micropinocytosis. While the nanocarriers with anionic features mainly tend to internalize through clathrin/caveolae-mediated endocytosis pathways (Foroozandeh and Aziz, 2018; Mazumdar et al., 2021). The TGA analysis further provided insights into the degradation patterns of the samples. As shown in Figure 3D, the bi-MIL-88B nanocarriers before the 5-FU incorporation exhibited two significant weight loss regions. The first weight loss below 280°C is attributed to the removal of coordinated solvent molecules in the structure (Gandara-Loe et al., 2020). The second considerable weight loss from 320°C to 480°C represents the decomposition of the organic linker and structural disintegration (Rojas et al., 2018). The drug-loaded 5-FU@bi-MIL-88B nanocarriers exhibited a weight loss pattern similar to the TGA of both 5-FU and bi-MIL-88B. The initial weight loss regions found in unloaded MOFs related to solvent molecules were not observed in the TGA of 5-FU@bi-MIL-88B, indicative of the pores filed with 5-FU molecules (Sheta et al., 2018). The initial weight loss till 320°C in the 5-FU@bi-MIL-88B is related to the decomposition of 5-FU molecules. In contrast, the second significant weight loss follows the pattern of linker decomposition similar to the unloaded MOFs. The 5-FU loaded nanocarriers coated with the FC exhibited a mixture of weight loss patterns identical to the TGA pattern of FC and 5-FU@bi-MIL-88B, which indicates the synthesis of FC-coated 5-FU@bi-MIL-88B composites (Nejadshafiee et al., 2019). Through the characterizations of SEM, FT-IR, PXRD, ZP, and TGA, the incorporation of the 5-FU and the subsequent coating by FC over the bi-MIL-88B nanocarriers was verified. Through the UV-Vis spectrophotometry analysis, the DLC and DLE of the nanocarriers were found to be 29.8% and 18.2%.

### 3.3 Drug release

The *in vitro* 5-FU release was investigated in two PBS mediums with variable pH mimicking the cancer cell environment (pH 5.2) and typical physiological environment (pH 7.4). The concentration of the 5-FU released from the nanocarriers was calculated by correlating the results with the 5-FU calibration curve in PBS (Supplementary Figure S9). The drug release behavior of 5-FU@bi-MIL-88B was compared with the FC-coated 5-FU@bi-MIL-88B-FC to examine the influence of the external coating on the release properties.

**FIGURE 4:**
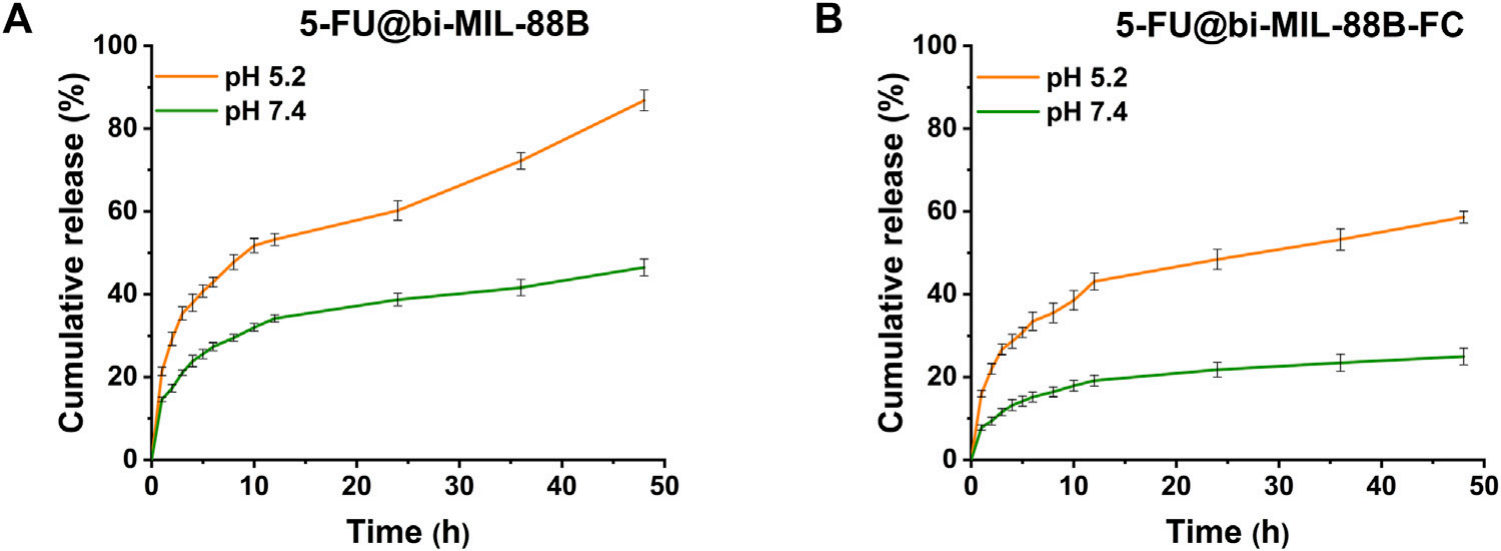
5-FU release pattern from **(A)** 5-FU@bi-MIL-88B; and **(B)** 5-FU@bi-MIL-88B-FC at different pHs (5.2 and 7.4).

The 5-FU release profiles of uncoated and coated bi-MIL-88B are shown in Figure 4. Generally, MOF-based drug delivery systems follow a two-step drug release pattern (Li et al., 2020). The first stage, rapid/burst release, is due to the drug molecules loosely bound to the surface of the nanocarriers. The quick release stage is followed by more sustained release related to nanocarriers’ structural modifications and departure of the drug molecules from the pores (Oh et al., 2015; Jiang et al., 2016). The 5-FU@bi-MIL-88B and 5-FU@bi-MIL-88B-FC followed a similar two-phase drug release kinetics pattern. A typical parabola of burst release during the first stage can be observed in all samples, with slight changes in both PBS (5.2 and 7.4). In the first 4 hours, 5-FU@bi-MIL-88B showed 23.8% and 37.9% of the 5-FU release in pH 5.2 and 7.4 (PBS). The drug release amounts reached 86.7% and 46.4% after 48 h in the second stage.

In contrast to the uncoated 5-FU@bi-MIL-88B, the FC-coated 5-FU@bi-MIL-88B-FC exhibited a much-controlled release kinetics of 5-FU in both PBS mediums simulating cancer microenvironment (pH = 5.2) and physiological environment (7.4). The FC-coated nanocarriers showed only 24.9% of drug release even after 48 h in the PBS of pH = 7.4, which could be beneficial to mitigate the unwanted toxicity of the drug to the normal cells. The lower release of the 5-FU from the 5-FU@bi-MIL-88B-FC nanocarriers under a physiological environment can be ascribed to the lower pKa (6.5) of the free amino groups in the CS. These groups lose their charge due to deprotonation at higher pHs and turn CS into an insoluble biopolymer shell. The insoluble coating act as a barrier to the premature release of the drug molecules. Moreover, the higher 5-FU release from the FC-coated nanocarriers in acidic PBS (pH = 5.2) compared to the physiological pH (7.4) is due to the protonation of the amine groups making the CS more soluble (Taghavi et al., 2017; Lu et al., 2019). Interestingly, the 5-FU@bi-MIL-88B-FC showed much sustained and slower release than their counterparts (5-FU@bi-MIL-88B) in the acidic pH (5.2). Only 58% of the 5-FU was released from the FC-coated nanocarriers compared to the uncoated ones, with 86% of the drug released during the same period. Due to the rapid degradation rate, chemotherapies based on free 5-FU administration lead to many issues, such as rapid cancer progression, metastasis and drug resistance (Leelakanok et al., 2018). Therefore, sustained release from the FC-coated 5-FU@bi-MIL-88B-FC could be helpful to overcome these challenges by a prolonged drug presence at the tumor site with target specificity of the carriers (Ali et al., 2023).

**FIGURE 5:**
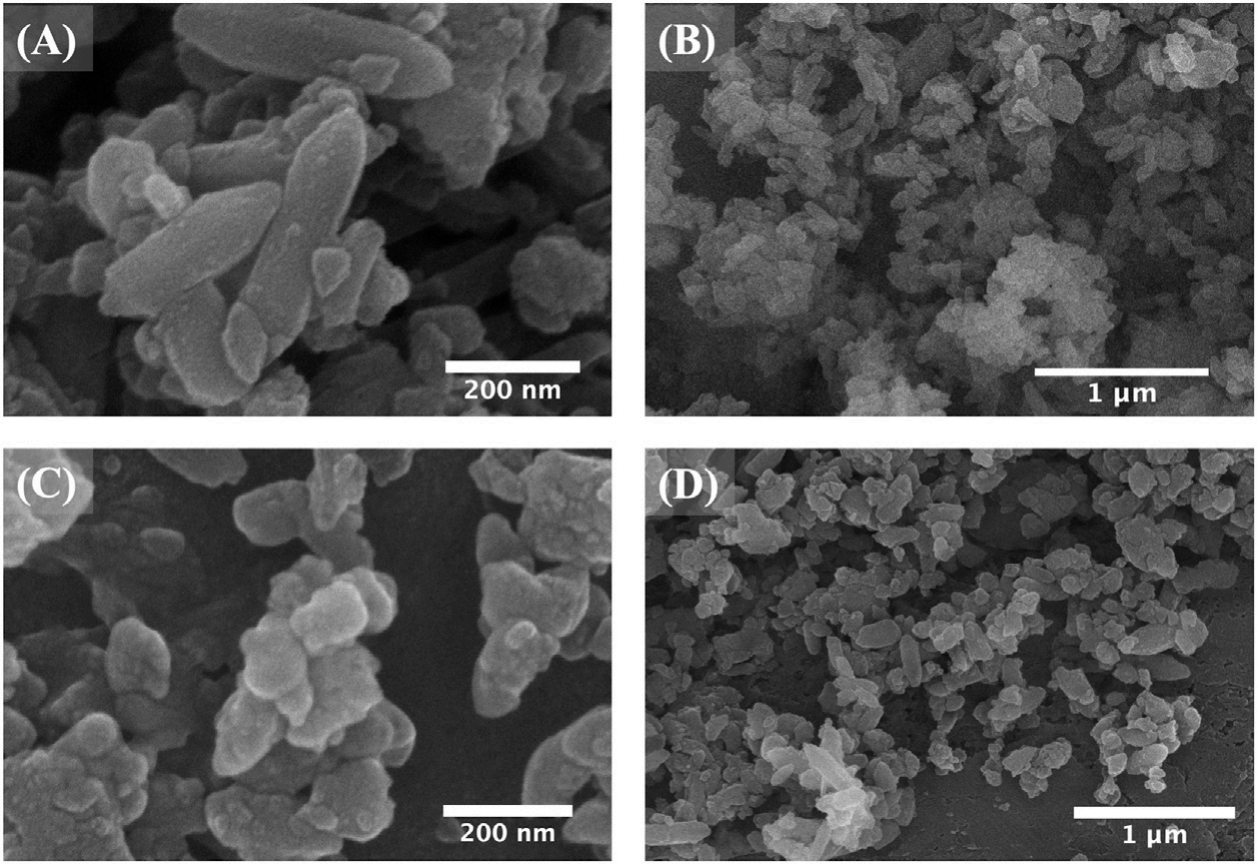
SEM images of 5-FU@bi-MIL-88B-FC after 4 days of immersion in PBS **(A, B)** of pH 7.4 and **(C, D)** pH 5.2.

The structural stability of 5-FU@bi-MIL-88B-FC was examined by immersing the samples in PBS of pH (5.2 and 7.4) for 4 days. According to the SEM images (Figures 5A, B), the nanocarriers immersed at a pH of 7.4 showed little or no difference in morphology, consistent with CS’s insoluble character at pHs above 6.5 (Liu et al., 2012). However, 5-FU@bi-MIL-88B-FC immersed in acidic media (pH = 5.2) showed complete degradation of the morphological traits resulting in distorted shape, indicating the drug release in acidic media due to structural breakdown (Figures 5C, D).

**FIGURE 6:**
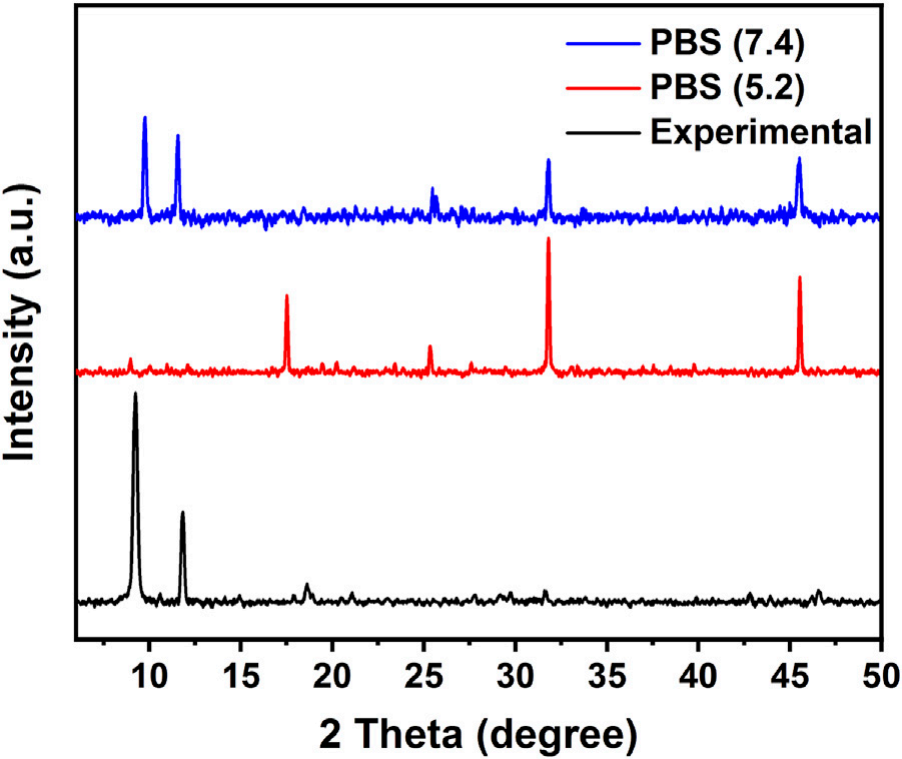
PXRD pattern of experimental bi-MIL-88B and immersed samples in PBS (pH 7.4 and 5.2).

PXRD analysis was obtained from these PBS (5.2 and 7.4) immersed samples further to analyze the structural alterations of nanocarriers under different pH. Figure 6., compares the PXRD pattern of PBS-immersed nanocarriers with the pure bi-MIL-88B. The nanocarriers soaked in PBS of pH 7.4 maintained most of the characteristic peaks reflected in the PXRD pattern of the pure bi-MIL-88B, supporting the good stability of MOF under physiological conditions also observed in SEM analysi. However, the PXRD pattern of the samples immersed under acidic pH (5.2) exhibited a loss of characteristic peaks of the parent MOFs indicating structural decomposition and instability. Moreover, the degradation of MOF in PBS could also be attributed to the strong affinity of phosphate ions present in the PBS towards the exposed metal sites in the MOF’s structure (Li et al., 2017). Evident from the pxrd pattern of samples immersed in acidic pH, the extra peaks found arround 17, 26, 32° and 46° (2theta) indicate the presence of Fe and Co phosphates due to their strong interaction (Beale and Sankar, 2002; Yuan et al., 2016).

### 3.4 *In Vitro* cytotoxicity and cellular uptake studies

**FIGURE 7:**
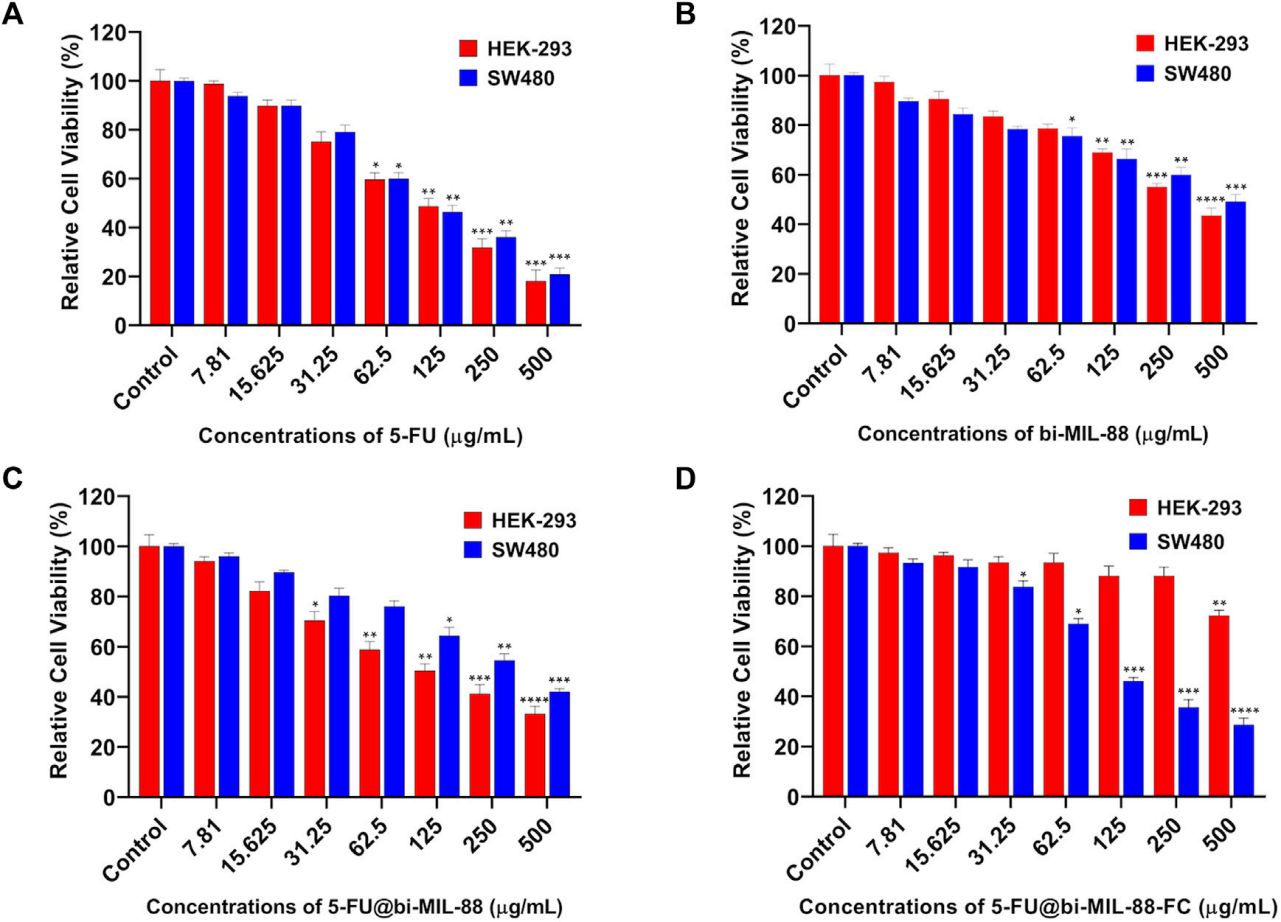
Cell viability results of HEK-293 and SW480 against different concentrations (7.81–500) of **(A)** 5-FU; **(B)** bi-MIL-88; **(C)** 5-FU@bi-MIL-88B and **(D)** 5-FU@bi-MIL-88B-FC. The degree of significance between the control and treatment groups for each cell line is denoted by ****p ≤ 0.0001, ***p ≤ 0.001, **p ≤ 0.01, and *p ≤ 0.05.

*In vitro*, the cytotoxicity profile of the samples was investigated to evaluate the efficacy of the FC-conjugated system for targeted 5-FU delivery. For this purpose, different concentrations (7.81–500 μg/mL) of 5-FU, bi-MIL-88B, 5-FU@bi-MIL-88B and 5-FU@bi-MIL-88B-FC were administered to the HEK-293 (FR-negative) and SW480 (FR-positive) cell lines. As seen in Figure 7A, 5-FU showed higher cytotoxic effects towards both cell lines due to its non-specific nature (Alvarez et al., 2012). The IC_50_ value of 5-FU and other treated agents are mentioned in Table 1. The unloaded and uncoated bi-MIL-88B MOFs showed considerable biocompatibility against HEK-293 cell lines with an IC_50_ value calculated at 342 μg/mL (Figure 7B). Moreover, 5-FU-loaded 5-FU@bi-MIL-88B nanocarriers exhibited concentration-dependent toxicity in HEK-293 and SW480 cells. The non-selective cytotoxicity behavior of 5-FU@bi-MIL-88B, if applied without FC coating, could lead to unwanted cytotoxicity against normal cells (Figure 7C) and cause failure of the whole system. The FC-coated 5-FU@bi-MIL-88B-FC exhibited selective toxicity against the FR-positive SW480 cells only with an IC_50_ of 136 μg/mL. A slightly higher IC_50_ value of FC-coated nanocarriers was observed compared to the free 5-FU against SW480 cells. It is because the free drug is readily available to the system to exert its effects during a short incubation time. In contrast, the encapsulated drug molecules are released slowly into the system and require more time to show their full efficacy (Gu et al., 2012). Moreover, 5-FU@bi-MIL-88B-FC showed very low toxicity towards the FR-negative cell lines (HEK-293), demonstrating the potential of the synthesized DDS to be effectively applied for targeted drug delivery against FR-positive cancer cell lines (Figure 7D).

**TABLE 1:** Estimated IC_50_ values of different treatment groups against HEK-293 and SW480 cells.

Treatment groups (μg/mL)				
Cell line	5-FU	bi-MIL-88B	5-FU@bi-MIL-88B	5-FU@bi-MIL-88B-FC
HEK-293	108	342	184	N.A
SW480	113	482	301	136

N.A: not accountable.

**FIGURE 8:**
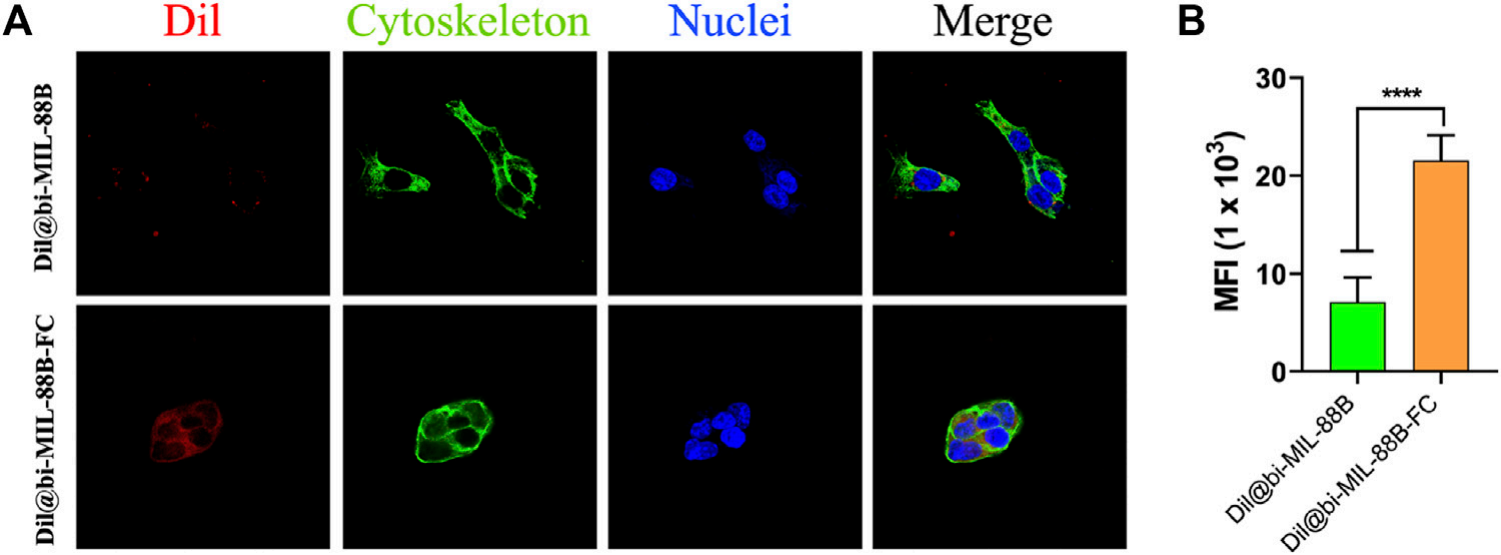
**(A)** Cellular internalization results of Dil@bi-MIL-88B (top) and Dil@bi-MIL-88B-FC (bottom) against SW480 cells visualized through CLSM. Red fluorescence (Dil), Green (Alexa fluor 488 stained cytoplasm), Blue (DAPI stained nuclei) and Merge (Overlay image); **(B)** Quantification of the MFI of Dil per cell via ImageJ. Data is shown in mean ± SD (n = 10). ****p < 0.0001.

To further support the observation of enhanced and selective toxicity of FC-coated nanocarriers against FR-positive SW480 cancer cells, the carbocyanine dye (Dil) labeled Dil@bi-MIL-88B and Dil@bi-MIL-88B-FC MOFs were used as a fluorescent probe. The Dil fluorescence intensity was measured using the excitation wavelength of 550 nm and an emission peak at 564 nm. The cellular uptake based on the Dil fluorescence intensity is shown in Figure 8A. The higher fluorescence intensity in cells treated with Dil@bi-MIL-88B-FC, compared to non-FC conjugated Dil@bi-MIL-88B, indicates the enhanced cellular uptake due to the FC shell. The cytoplasm was stained with alexa fluor 488 phalloidin, and the nucleus was stained with DAPI. Moreover, the increaed in the mean fluorescence intensity (MFI) by 1.8 to 2.4- fold for cells treated with Dil@bi-MIL-88B-FC compared to Dil@bi-MIL-88B further corroborated to the excellent cellular uptake of FC functionalized nanocarriers. These results suggest that the FC coating facilitates folate receptor-mediated cellular uptake and is essential in developing targeted DDS (Stella et al., 2000; Song et al., 2013).

### 3.5 Peroxidase-like activity

**SCHEME 2:**
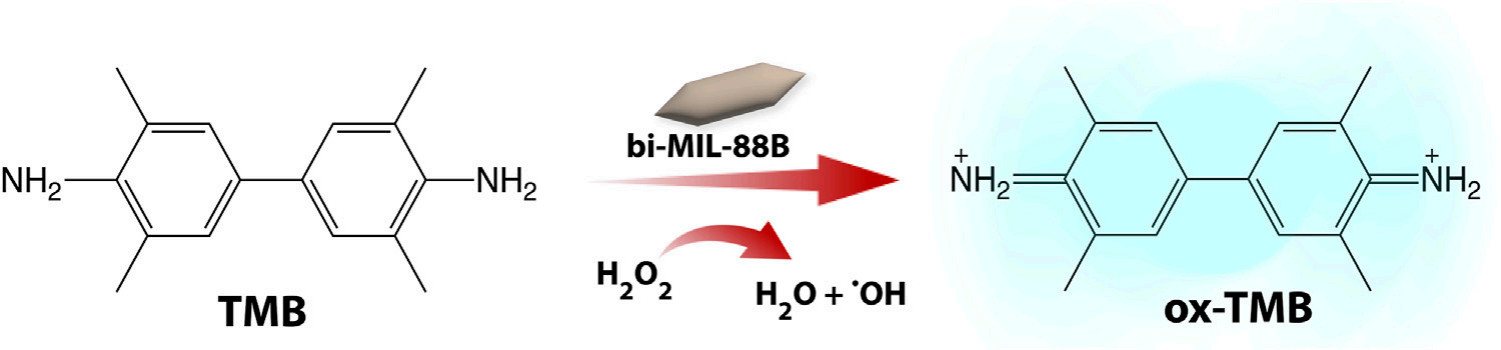
The peroxidase-like activity of bi-MIL-88B nanocarriers.

Inspired by the peroxidase (POD) like activity of the different transition metals such as Fe, Mn, Cu, and Co, and their use in chemodynamic therapy, we examined the POD activity of our nanocarriers through the TMB oxidation test (Scheme 2) (Bokare and Choi, 2014). Due to their altered metabolic pathways, the cancer cells are known to have higher levels of reactive oxygen species (H_2_O_2_, ^1^O_2_, ^•^OH) production (Giacosa et al., 2021). This over-expressed ROS production is utilized by cancer cells for various purposes, such as drug resistance, tumor pathogenesis, and metastasis (Ishikawa et al., 2008; Chun et al., 2021). The bi-MIL-88B MOFs, due to their trinuclear oxo cluster with terminal coordinatively unsaturated sites (CUS), are capable of decomposing H_2_O into highly toxic ^•^OH radicals (Xiao et al., 2023). These ^•^OH radicals are highly potent and can oxidize any macromolecules that come in contact with them (Li H. et al., 2022).

**FIGURE 9:**
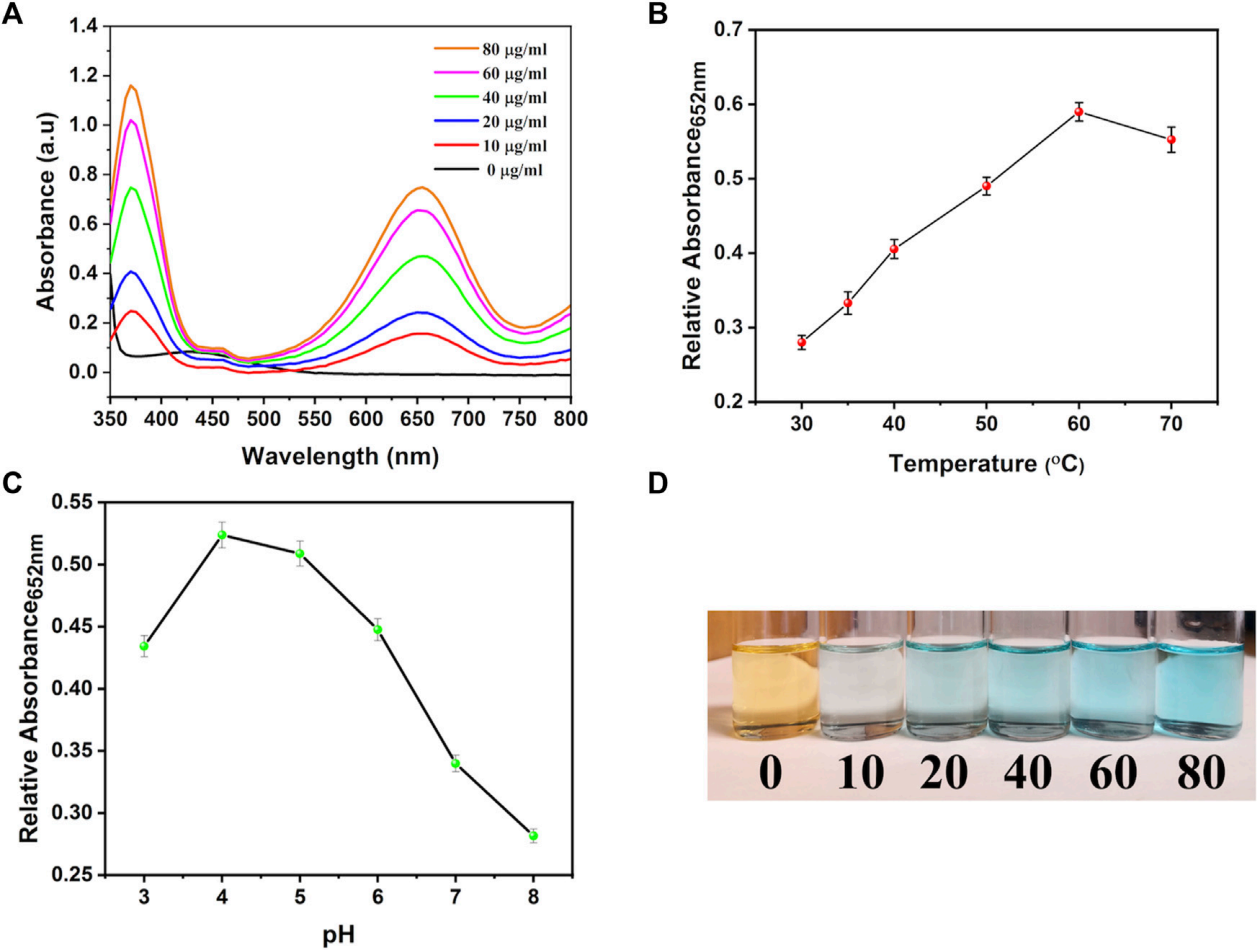
**(A)** TMB oxidation with different concentrations of bi-MIL-88B; **(B)** Effect of temperature and **(C)** pH on the POD activity of bi-MIL-88B; **(D)** Digital photograph of TMB color change during the oxidation process.

Similar results were observed in the current study; when the system contained only H_2_O_2_ and TMB, no catalytic reaction was observed regarding TMB oxidation (Figure 9A). However, by adding different concentrations of the bi-MIL-88B, the colorless TMB started to convert into a blue-colored oxidized form (detected at 652 nm wavelength), indicating the POD potential. Furthermore, mechanistic studies were performed to evaluate the performance of bi-MIL-88B nanocarriers under variable pH and temperature. As seen in Figure 9B, an increase in the pH from 4 to 8 reduced the production of oxidized TMB, indicating our nanocarriers’ safety at physiological pH (7.4). Higher catalytic activity in the samples in an acidic pH medium suggested the cancer cell-specific POD performance. The catalytic performance also increased by increasing the system’s temperature (Figure 9C). The increased activity with higher temperatures benefits our developed DDS, as cancer cells usually have higher internal temperatures than normal cells (Vahed et al., 2017). The enhanced POD performance under rising temperature can be related to the altered entropy of the reaction constant and reduced activation energy needed for the catalytic reaction (Åqvist et al., 2017). Based on the multidimensional therapeutic ability, 5-FU@bi-MIL-88B-FC present excellent potential in the field of multifaceted targeted therapies. Some of the MOF based carriers functionalized through FA for targeted therapies are mentioned in Table 2.

**TABLE 2:** List of reported MOF based nanocarriers functionalized with folic acid for targeted therapy.

MOF	Functionalization	Drug	Loading capacity	Stimuli	Therapy	Reference
ZIF-67@ZIF-8	Iron oxide, FA	Quercetin	50 wt%	pH	CT, CDT	Pandit et al. (2022)
BioMOF-101	FA	Curcumin	99.42 wt%	pH	CT	Alves et al. (2023)
MOF-808	FA - CS	Quercetin	43 wt%	pH	CT	Parsaei and Akhbari (2022b)
Zr-MOF	FA	Bufalin	17.4 wt%	pH, GSH	CT	Zeng et al. (2022)
PCN-224	FA	Camptothecin, Doxorubicin	10,7 and 6.8 wt%	pH	CT	Xie et al. (2022)
Fe-MIL-88@ZIF-8	FA	Doxorubicin, MnOx	43.2 wt%	pH	CT, CDT	Zeng et al. (2021)
Zn-MOF	FA - CS	Methotrexate	78 wt%	pH	CT	Khatibi et al. (2022)
UiO-66-NH2	FA	Oxaliplatin	29.3 wt%	pH	CT	Hashemzadeh et al. (2021)
ZIF-8	FA	miR-491–59		pH	Gene regulation	Ju et al. (2021)
UiO-66	FA– Pluronic F127 and SiO_2_	Doxorubicin	5.6 wt%	pH	CT	Trushina et al. (2022)
Fe-MOF-5-NH2	FA, 5-FAM	5-FU	35 wt%	pH	CT	Gao et al. (2019)
Bi-MIL-88B	FA—CS	5-FU	29.8 wt%	pH	CT, CDT	This study

As it is crucial for the effectiveness of any therapy that drug accumulation should be in the target sites rather off-target (Farooq et al., 2019; Saddozai et al., 2020). In case of leakage to healthy tissue, adverse effects in terms of cellular cytotoxicity could be observed, leading to severe complications (Torchilin, 2010; He et al., 2020). Several factors, such as the physicochemical properties of drug molecules and tumor biology, can affect passive targeting. Therefore, these issues can be addressed by functionalizing drug-loaded nanocarriers with targeting ligands (Attia et al., 2019; Tesauro et al., 2019). FA is the most commonly used ligand for MOFs’ surface functionalization to obtain FR-receptor targeting (Muhamad et al., 2018). In addition to active targeting, MOF-based nanocarriers utilizing intrinsic components of TME to generate ROS stress for a synergistic therapeutic effect along with chemotherapy present a new class of intelligent nanomaterials for efficient anticancer properties (Wang C. et al., 2022; Wang et al., 2022c; Liang et al., 2023). In this regard, bi-MIL-88B-FC can be effectively utilized as a potential DDS for multidimensional targeted chemotherapy and chemodynamic therapy based on mechanistic insights into the catalytic performance and drug release kinetics.

## 4 Conclusion

In this study, 5-FU@bi-MIL-88B-FC nanocarriers were synthesized for tumor-specific targeted drug delivery. The nanocarriers presented a higher 5-FU loading capacity of 29.8 wt%. Moreover, surface modification through the FA conjugated CS (FC) endowed these carriers with exceptional cell targeting and sustained drug release properties. The presence of an extra polymer coating provided a gated effect in improving the controlled release of the loaded drug and evasion of premature leakage. The 5-FU@bi-MIL-88B-FC exhibited pH-responsive drug release with higher concentrations of the 5-FU released under the tumor-mimicking environment (pH 5.2). The cytotoxicity profile and folate receptor-mediated cellular uptake was investigated against HEK-293 (FR-negative) and cancer SW480 (FR-positive). The results showed FR-positive cancer cell-specific cytotoxic effects of 5-FU@bi-MIL-88B-FC against the SW480 cells with sufficient internalization efficacy. Moreover, the peroxidase-like activity due to the catalytic sites provides these nanocarriers an extra feature to be tested for a full-fledged multidimensional anticancer therapy. The sufficient short-time stability, stimuli-responsive drug release, POD mimicking character and active targeting of FR-positive tumor cells with FA binding make these nanocarriers promising DDS for multifunctional tumor therapy.

## Data Availability

The original contributions presented in the study are included in the article/Supplementary Material, further inquiries can be directed to the corresponding authors.

## References

[B1] Abánades Lázaro I. Wells C. J. Forgan R. S. (2020). Multivariate modulation of the Zr MOF UiO‐66 for defect‐controlled combination anticancer drug delivery. Angew. Chem. 132, 5211–5217. 10.1002/anie.201915848 PMC7154787 31950568

[B2] Abbas M. Maceda A. M. Xiao Z. Zhou H.-C. Balkus K. J. (2023). Transformation of a copper-based metal–organic polyhedron into a mixed linker MOF for CO 2 capture. Dalton Trans. 52, 4415–4422. 10.1039/d2dt04162f 36916445

[B3] Adegoke K. A. Maxakato N. W. (2021). Porous metal-organic framework (MOF)-based and MOF-derived electrocatalytic materials for energy conversion. Mater. Today Energy 21, 100816. 10.1016/j.mtener.2021.100816

[B4] Aibani N. Rai R. Patel P. Cuddihy G. Wasan E. K. (2021). Chitosan nanoparticles at the biological interface: implications for drug delivery. Pharmaceutics 13, 1686. 10.3390/pharmaceutics13101686 34683979 PMC8540112

[B5] Akbar M. U. Badar M. Zaheer M. (2022). Programmable drug release from a dual-stimuli responsive magnetic metal–organic framework. ACS omega 7, 32588–32598. 10.1021/acsomega.2c04144 36120053 PMC9475617

[B6] Akbarzadeh I. Shayan M. Bourbour M. Moghtaderi M. Noorbazargan H. Eshrati Yeganeh F. (2021). Preparation, optimization and *in-vitro* evaluation of curcumin-loaded niosome@ calcium alginate nanocarrier as a new approach for breast cancer treatment. Biology 10, 173. 10.3390/biology10030173 33652630 PMC7996962

[B7] Ali A. Madni A. Shah H. Jamshaid T. Jan N. Khan S. (2023). Solid lipid-based nanoparticulate system for sustained release and enhanced *in-vitro* cytotoxic effect of 5-fluorouracil on skin Melanoma and squamous cell carcinoma. Plos one 18, e0281004. 10.1371/journal.pone.0281004 36854019 PMC9974133

[B8] Al-Nemrawi N. K. Altawabeyeh R. M. Darweesh R. S. (2022). Preparation and characterization of docetaxel-PLGA nanoparticles coated with folic acid-chitosan conjugate for cancer treatment. J. Pharm. Sci. 111, 485–494. 10.1016/j.xphs.2021.10.034 34728172

[B9] Altintas C. Erucar I. Keskin S. (2022). MOF/COF hybrids as next generation materials for energy and biomedical applications. CrystEngComm 24, 7360–7371. 10.1039/d2ce01296k 36353708 PMC9620950

[B10] Alvarez P. Marchal J. A. Boulaiz H. Carrillo E. Vélez C. Rodríguez-Serrano F. (2012). 5-Fluorouracil derivatives: A patent review. Expert Opin. Ther. Pat. 22, 107–123. 10.1517/13543776.2012.661413 22329541

[B11] Alves R. C. Quijia C. R. da Silva P. B. Faria R. S. Morais A. A. C. Morais J. A. V. (2023). Folic acid-conjugated curcumin-loaded bioMOF-101 for breast cancer therapy. J. Drug Deliv. Sci. Technol. 86, 104702. 10.1016/j.jddst.2023.104702

[B12] Anisimov R. A. Gorin D. A. Abalymov A. A. (2022). 3D cell spheroids as A tool for evaluating the effectiveness of carbon nanotubes as A drug delivery and photothermal therapy agents. C 8, 56. 10.3390/c8040056

[B13] Åqvist J. Kazemi M. Isaksen G. V. Brandsdal B. O. (2017). Entropy and enzyme catalysis. Accounts Chem. Res. 50, 199–207. 10.1021/acs.accounts.6b00321 28169522

[B14] Attia M. F. Anton N. Wallyn J. Omran Z. Vandamme T. F. (2019). An overview of active and passive targeting strategies to improve the nanocarriers efficiency to tumour sites. J. Pharm. Pharmacol. 71, 1185–1198. 10.1111/jphp.13098 31049986

[B15] Bäumer N. Tiemann J. Scheller A. Meyer T. Wittmann L. Suburu M. E. G. (2022). Targeted siRNA nanocarrier: a platform technology for cancer treatment. Oncogene 41, 2210–2224. 10.1038/s41388-022-02241-w 35220407 PMC8993695

[B16] Beale A. M. Sankar G. (2002). Following the structural changes in iron phosphate catalysts by *in situ* combined XRD/QuEXAFS technique. J. Mater. Chem. 12, 3064–3072. 10.1039/b204059j

[B17] Bokare A. D. Choi W. (2014). Review of iron-free Fenton-like systems for activating H2O2 in advanced oxidation processes. J. Hazard. Mater. 275, 121–135. 10.1016/j.jhazmat.2014.04.054 24857896

[B18] Cai X. Lin J. Pang M. (2016). Facile synthesis of highly uniform Fe-MIL-88B particles. Cryst. Growth Des. 16, 3565–3568. 10.1021/acs.cgd.6b00313

[B19] Cao D. Sha Q. Wang J. Li J. Ren J. Shen T. (2022). Advanced anode materials for sodium-ion batteries: confining polyoxometalates in flexible metal–organic frameworks by the “breathing effect”. ACS Appl. Mater. Interfaces 14, 22186–22196. 10.1021/acsami.2c04077 35510903

[B20] Chang C.-T. Ho T.-Y. Lin H. Liang J.-A. Huang H.-C. Li C.-C. (2012). 5-Fluorouracil induced intestinal mucositis via nuclear factor-κB activation by transcriptomic analysis and *in vivo* bioluminescence imaging. PloS one 7, e31808. 10.1371/journal.pone.0031808 22412841 PMC3296709

[B21] Chanphai P. Konka V. Tajmir-Riahi H. (2017). Folic acid–chitosan conjugation: A new drug delivery tool. J. Mol. Liq. 238, 155–159. 10.1016/j.molliq.2017.04.132

[B22] Chen Q. Wang X. Chen F. Zhang Q. Dong B. Yang H. (2011). Functionalization of upconverted luminescent NaYF4: Yb/Er nanocrystals by folic acid–chitosan conjugates for targeted lung cancer cell imaging. J. Mater. Chem. 21, 7661–7667. 10.1039/c0jm04468g

[B23] Chen C. Yu Y. Wang X. Shi P. Wang Y. Wang P. (2017). Manipulation of pH-Sensitive interactions between podophyllotoxin-chitosan for enhanced controlled drug release. Int. J. Biol. Macromol. 95, 451–461. 10.1016/j.ijbiomac.2016.11.053 27867056

[B24] Chen Z.-J. Yang S.-C. Liu X.-L. Gao Y. Dong X. Lai X. (2020). Nanobowl-supported liposomes improve drug loading and delivery. Nano Lett. 20, 4177–4187. 10.1021/acs.nanolett.0c00495 32431154

[B25] Chowdhuri A. R. Laha D. Pal S. Karmakar P. Sahu S. K. (2016). One-pot synthesis of folic acid encapsulated upconversion nanoscale metal organic frameworks for targeting, imaging and pH responsive drug release. Dalton Trans. 45, 18120–18132. 10.1039/c6dt03237k 27785489

[B26] Chun K.-S. Kim D.-H. Surh Y.-J. (2021). Role of reductive versus oxidative stress in tumor progression and anticancer drug resistance. Cells 10, 758. 10.3390/cells10040758 33808242 PMC8065762

[B27] Das P. K. Islam F. Lam A. K. (2020). The roles of cancer stem cells and therapy resistance in colorectal carcinoma. Cells 9, 1392. 10.3390/cells9061392 32503256 PMC7348976

[B28] Di X. Pei Z. Pei Y. James T. D. (2023). Tumor microenvironment-oriented MOFs for chemodynamic therapy. Coord. Chem. Rev. 484, 215098. 10.1016/j.ccr.2023.215098

[B29] Dibden A. Offman J. Duffy S. W. Gabe R. (2020). Worldwide review and meta-analysis of cohort studies measuring the effect of mammography screening programmes on incidence-based breast cancer mortality. Cancers 12, 976. 10.3390/cancers12040976 32326646 PMC7226343

[B30] El Leithy E. S. Abdel-Bar H. M. Ali R. A.-M. (2019). Folate-chitosan nanoparticles triggered insulin cellular uptake and improved *in vivo* hypoglycemic activity. Int. J. Pharm. 571, 118708. 10.1016/j.ijpharm.2019.118708 31593805

[B31] Farooq M. A. Aquib M. Farooq A. Haleem Khan D. Joelle Maviah M. B. Sied Filli M. (2019). Recent progress in nanotechnology-based novel drug delivery systems in designing of cisplatin for cancer therapy: an overview. Artif. Cells, Nanomed. Biotechnol. 47, 1674–1692. 10.1080/21691401.2019.1604535 31066300

[B32] Foroozandeh P. Aziz A. A. (2018). Insight into cellular uptake and intracellular trafficking of nanoparticles. Nanoscale Res. Lett. 13, 1–12. 10.1186/s11671-018-2728-6 30361809 PMC6202307

[B33] Gandara-Loe J. Souza B. E. Missyul A. Giraldo G. Tan J.-C. Silvestre-Albero J. (2020). MOF-based polymeric nanocomposite films as potential materials for drug delivery devices in ocular therapeutics. ACS Appl. Mater. Interfaces 12, 30189–30197. 10.1021/acsami.0c07517 32530261

[B34] Gao X. Cui R. Song L. Liu Z. (2019). Hollow structural metal–organic frameworks exhibit high drug loading capacity, targeted delivery and magnetic resonance/optical multimodal imaging. Dalton Trans. 48, 17291–17297. 10.1039/c9dt03287h 31714562

[B35] Ge X. Wong R. Anisa A. Ma S. (2022). Recent development of metal-organic framework nanocomposites for biomedical applications. Biomaterials 281, 121322. 10.1016/j.biomaterials.2021.121322 34959029

[B36] Giacosa S. Pillet C. Séraudie I. Guyon L. Wallez Y. Roelants C. (2021). Cooperative blockade of CK2 and ATM kinases drives apoptosis in VHL-deficient renal carcinoma cells through ROS overproduction. Cancers 13, 576. 10.3390/cancers13030576 33540838 PMC7867364

[B37] Gu P. F. Xu H. Sui B. W. Gou J. X. Meng L. K. Sun F. (2012). Polymeric micelles based on poly (ethylene glycol) block poly (racemic amino acids) hybrid polypeptides: conformation-facilitated drug-loading behavior and potential application as effective anticancer drug carriers. Int. J. Nanomed. 7, 109–122. 10.2147/IJN.S27475 PMC3260955 22275827

[B38] Guo J. Yu Z. Das M. Huang L. (2020). Nano codelivery of oxaliplatin and folinic acid achieves synergistic chemo-immunotherapy with 5-fluorouracil for colorectal cancer and liver metastasis. ACS Nano 14, 5075–5089. 10.1021/acsnano.0c01676 32283007

[B39] Hashemzadeh A. Amerizadeh F. Asgharzadeh F. Darroudi M. Avan A. Hassanian S. M. (2021). Delivery of oxaliplatin to colorectal cancer cells by folate-targeted UiO-66-NH2. Toxicol. Appl. Pharmacol. 423, 115573. 10.1016/j.taap.2021.115573 33991535

[B40] He Z. Zhang Y. Feng N. (2020). Cell membrane-coated nanosized active targeted drug delivery systems homing to tumor cells: A review. Mater. Sci. Eng. C 106, 110298. 10.1016/j.msec.2019.110298 31753336

[B41] Hesse D. Badar M. Bleich A. Smoczek A. Glage S. Kieke M. (2013). Layered double hydroxides as efficient drug delivery system of ciprofloxacin in the middle ear: an animal study in rabbits. J. Mater. Sci. Mater. Med. 24, 129–136. 10.1007/s10856-012-4769-1 23053799

[B42] Horcajada P. Serre C. Maurin G. Ramsahye N. A. Balas F. Vallet-Regi M. (2008). Flexible porous metal-organic frameworks for a controlled drug delivery. J. Am. Chem. Soc. 130, 6774–6780. 10.1021/ja710973k 18454528

[B43] Horcajada P. Salles F. Wuttke S. Devic T. Heurtaux D. Maurin G. (2011). How linker’s modification controls swelling properties of highly flexible iron (III) dicarboxylates MIL-88. J. Am. Chem. Soc. 133, 17839–17847. 10.1021/ja206936e 21950795

[B44] Horcajada P. Gref R. Baati T. Allan P. K. Maurin G. Couvreur P. (2012). Metal–organic frameworks in biomedicine. Chem. Rev. 112, 1232–1268. 10.1021/cr200256v 22168547

[B45] Hu Y. Ke L. Chen H. Zhuo M. Yang X. Zhao D. (2017). Natural material-decorated mesoporous silica nanoparticle container for multifunctional membrane-controlled targeted drug delivery. Int. J. Nanomed. 12, 8411–8426. 10.2147/IJN.S148438 PMC5702528 29200852

[B46] İnce İ. Yıldırım Y. Güler G. Medine E. İ. Ballıca G. Kuşdemir B. C. (2020). Synthesis and characterization of folic acid-chitosan nanoparticles loaded with thymoquinone to target ovarian cancer cells. J. Radioanal. Nucl. Chem. 324, 71–85. 10.1007/s10967-020-07058-z

[B47] Iqbal B. Saleem M. Arshad S. N. Rashid J. Hussain N. Zaheer M. (2019). One‐pot synthesis of heterobimetallic metal–organic frameworks (MOFs) for multifunctional catalysis. Chemistry–A Eur. J. 25, 10490–10498. 10.1002/chem.201901939 31163099

[B48] Iqbal B. Laybourn A. Zaheer M. (2021). Size-controlled synthesis of spinel nickel ferrite nanorods by thermal decomposition of a bimetallic Fe/Ni-MOF. Ceram. Int. 47, 12433–12441. 10.1016/j.ceramint.2021.01.100

[B49] Ishihara K. Kitagawa T. Inoue Y. (2015). Initial cell adhesion on well-defined surface by polymer brush layers with varying chemical structures. ACS Biomaterials Sci. Eng. 1, 103–109. 10.1021/ab500048w

[B50] Ishikawa K. Takenaga K. Akimoto M. Koshikawa N. Yamaguchi A. Imanishi H. (2008). ROS-generating mitochondrial DNA mutations can regulate tumor cell metastasis. Science 320, 661–664. 10.1126/science.1156906 18388260

[B51] Jiang K. Zhang L. Hu Q. Zhao D. Xia T. Lin W. (2016). Pressure controlled drug release in a Zr-cluster-based MOF. J. Mater. Chem. B 4, 6398–6401. 10.1039/c6tb01756h 32263448

[B52] Ju G. Liu B. Ji M. Jin R. Xu X. Xiao Y. (2021). Folic acid–modified miR-491-5p–Loaded ZIF-8 nanoparticles inhibit castration-resistant prostate cancer by regulating the expression of EPHX1. Front. Bioeng. Biotechnol. 9, 706536. 10.3389/fbioe.2021.706536 34881229 PMC8645958

[B53] Karimi Alavijeh R. Akhbari K. (2020). Biocompatible MIL-101 (Fe) as a smart carrier with high loading potential and sustained release of curcumin. Inorg. Chem. 59, 3570–3578. 10.1021/acs.inorgchem.9b02756 32091212

[B54] Khatibi Z. Kazemi N. M. Khaleghi S. (2022). Targeted and biocompatible NMOF as efficient nanocomposite for delivery of methotrexate to colon cancer cells. J. Drug Deliv. Sci. Technol. 73, 103441. 10.1016/j.jddst.2022.103441

[B124] Leelakanok N. Geary S. Salem A. (2018). Fabrication and use of poly (d, l-lactide-co-glycolide)-based formulations designed for modified release of 5-fluorouracil. Journal of pharmaceutical sciences 107 (2), 513–528. 29045885 10.1016/j.xphs.2017.10.012 PMC5768463

[B56] Li L. Xiang S. Cao S. Zhang J. Ouyang G. Chen L. (2013). A synthetic route to ultralight hierarchically micro/mesoporous Al (III)-carboxylate metal-organic aerogels. Nat. Commun. 4, 1774. 10.1038/ncomms2757 23653186 PMC3644084

[B57] Li X. Lachmanski L. Safi S. Sene S. Serre C. Grenèche J.-M. (2017). New insights into the degradation mechanism of metal-organic frameworks drug carriers. Sci. Rep. 7, 13142. 10.1038/s41598-017-13323-1 29030570 PMC5640595

[B58] Li L. Han S. Zhao S. Li X. Liu B. Liu Y. (2020). Chitosan modified metal–organic frameworks as a promising carrier for oral drug delivery. RSC Adv. 10, 45130–45138. 10.1039/d0ra08459j 35516251 PMC9058666

[B59] Li Y. Jiang Y. Zheng Z. Du N. Guan S. Guo W. (2022a). Co‐Delivery of precisely prescribed multi‐prodrug combination by an engineered nanocarrier enables efficient individualized cancer chemotherapy. Adv. Mater. 34, 2110490. 10.1002/adma.202110490 35044690

[B60] Li H. Zhang Y. Liang L. Song J. Wei Z. Yang S. (2022b). Doxorubicin-loaded metal-organic framework nanoparticles as acid-activatable hydroxyl radical nanogenerators for enhanced chemo/chemodynamic synergistic therapy. Materials 15, 1096. 10.3390/ma15031096 35161041 PMC8838206

[B61] Liang J. Zhang W. Wang J. Li W. Ge F. Jin W. (2023). Development of the Cu/ZIF-8 MOF acid-sensitive nanocatalytic platform capable of chemo/chemodynamic therapy with improved anti-tumor efficacy. ACS Omega 8, 19402–19412. 10.1021/acsomega.3c00269 37305251 PMC10249029

[B62] Liu H. Wang C. Zou S. Wei Z. Tong Z. (2012). Simple, reversible emulsion system switched by pH on the basis of chitosan without any hydrophobic modification. Langmuir 28, 11017–11024. 10.1021/la3021113 22762435

[B63] Liu Y. L. Zhao X. J. Yang X. X. Li Y. F. (2013). A nanosized metal-organic framework of Fe-MIL-88NH₂ as a novel peroxidase mimic used for colorimetric detection of glucose. Analyst 138, 4526–4531. 10.1039/c3an00560g 23775015

[B64] Liu Q. Cong H. Deng H. (2016). Deciphering the spatial arrangement of metals and correlation to reactivity in multivariate metal–organic frameworks. J. Am. Chem. Soc. 138, 13822–13825. 10.1021/jacs.6b08724 27701854

[B65] Longley D. B. Harkin D. P. Johnston P. G. (2003). 5-fluorouracil: mechanisms of action and clinical strategies. Nat. Rev. cancer 3, 330–338. 10.1038/nrc1074 12724731

[B66] Lu B. Lv X. Le Y. (2019). Chitosan-modified PLGA nanoparticles for control-released drug delivery. Polymers 11, 304. 10.3390/polym11020304 30960288 PMC6419218

[B67] Lv G. Qiu L. Liu G. Wang W. Li K. Zhao X. (2016). pH sensitive chitosan-mesoporous silica nanoparticles for targeted delivery of a ruthenium complex with enhanced anticancer effects. Dalton Trans. 45, 18147–18155. 10.1039/c6dt03783f 27785492

[B68] Lyu L. Zhang L. He G. He H. Hu C. (2017). Selective H 2 O 2 conversion to hydroxyl radicals in the electron-rich area of hydroxylated CgC 3 N 4/CuCo–Al 2 O 3. J. Mater. Chem. A 5, 7153–7164. 10.1039/c7ta01583f

[B69] Ma M. Bétard A. Weber I. Al-Hokbany N. S. Fischer R. A. Metzler-Nolte N. (2013). Iron-based metal–organic frameworks MIL-88B and NH2-MIL-88B: high quality microwave synthesis and solvent-induced lattice “breathing”. Cryst. Growth Des. 13, 2286–2291. 10.1021/cg301738p

[B70] Manzari M. T. Shamay Y. Kiguchi H. Rosen N. Scaltriti M. Heller D. A. (2021). Targeted drug delivery strategies for precision medicines. Nat. Rev. Mater. 6, 351–370. 10.1038/s41578-020-00269-6 34950512 PMC8691416

[B71] Mazumdar S. Chitkara D. Mittal A. (2021). Exploration and insights into the cellular internalization and intracellular fate of amphiphilic polymeric nanocarriers. Acta Pharm. Sin. B 11, 903–924. 10.1016/j.apsb.2021.02.019 33996406 PMC8105776

[B72] Muhamad N. Plengsuriyakarn T. Na-Bangchang K. (2018). Application of active targeting nanoparticle delivery system for chemotherapeutic drugs and traditional/herbal medicines in cancer therapy: A systematic review. Int. J. nanomedicine 13, 3921–3935. 10.2147/IJN.S165210 30013345 PMC6038858

[B73] Nejadshafiee V. Naeimi H. Goliaei B. Bigdeli B. Sadighi A. Dehghani S. (2019). Magnetic bio-metal–organic framework nanocomposites decorated with folic acid conjugated chitosan as a promising biocompatible targeted theranostic system for cancer treatment. Mater. Sci. Eng. C 99, 805–815. 10.1016/j.msec.2019.02.017 30889755

[B74] Nemati M. Bani F. Sepasi T. Zamiri R. E. Rasmi Y. Kahroba H. (2021). Unraveling the effect of breast cancer patients’ plasma on the targeting ability of folic acid-modified chitosan nanoparticles. Mol. Pharm. 18, 4341–4353. 10.1021/acs.molpharmaceut.1c00525 34779630

[B75] Oh H. Li T. An J. (2015). Drug release properties of a series of adenine‐based metal–organic frameworks. Chemistry–A Eur. J. 21, 17010–17015. 10.1002/chem.201501560 26403522

[B76] Oh J. Y. Choi E. Jana B. Go E. M. Jin E. Jin S. (2023). Protein‐precoated surface of metal‐organic framework nanoparticles for targeted delivery. Small 2023, 2300218. 10.1002/smll.202300218 36864579

[B77] Okur S. Qin P. Chandresh A. Li C. Zhang Z. Lemmer U. (2021). An enantioselective e‐nose: an array of nanoporous homochiral MOF films for stereospecific sensing of chiral odors. Angew. Chem. Int. Ed. 60, 3566–3571. 10.1002/anie.202013227 PMC7898876 33156561

[B78] Pandit P. Bhagat S. Rananaware P. Mohanta Z. Kumar M. Tiwari V. (2022). Iron oxide nanoparticle encapsulated; folic acid tethered dual metal organic framework-based nanocomposite for MRI and selective targeting of folate receptor expressing breast cancer cells. Microporous Mesoporous Mater. 340, 112008. 10.1016/j.micromeso.2022.112008

[B79] Parsaei M. Akhbari K. (2022a). Smart multifunctional UiO-66 metal–organic framework nanoparticles with outstanding drug-loading/release potential for the targeted delivery of quercetin. Inorg. Chem. 61, 14528–14543. 10.1021/acs.inorgchem.2c00743 36074039

[B80] Parsaei M. Akhbari K. (2022b). Synthesis and application of MOF-808 decorated with folic acid-conjugated chitosan as a strong nanocarrier for the targeted drug delivery of quercetin. Inorg. Chem. 61, 19354–19368. 10.1021/acs.inorgchem.2c03138 36383693

[B81] Peers S. Montembault A. Ladavière C. (2020). Chitosan hydrogels for sustained drug delivery. J. Control. Release 326, 150–163. 10.1016/j.jconrel.2020.06.012 32562854

[B82] Peng L. Asgari M. Mieville P. Schouwink P. Bulut S. Sun D. T. (2017). Using predefined M3 (μ3-O) clusters as building blocks for an isostructural series of metal–organic frameworks. ACS Appl. Mater. Interfaces 9, 23957–23966. 10.1021/acsami.7b06041 28650146

[B83] Rana I. Oh J. Baig J. Moon J. H. Son S. Nam J. (2023). Nanocarriers for cancer nano-immunotherapy. Drug Deliv. Transl. Res. 13, 1936–1954. 10.1007/s13346-022-01241-3 36190661 PMC9528883

[B84] Rojas S. Colinet I. Cunha D. Hidalgo T. Salles F. Serre C. (2018). Toward understanding drug incorporation and delivery from biocompatible metal–organic frameworks in view of cutaneous administration. ACS Omega 3, 2994–3003. 10.1021/acsomega.8b00185 29623304 PMC5879486

[B85] Saddozai U. A. K. Wang F. Cheng Y. Lu Z. Akbar M. U. Zhu W. (2020). Gene expression profile identifies distinct molecular subtypes and potential therapeutic genes in Merkel cell carcinoma. Transl. Oncol. 13, 100816. 10.1016/j.tranon.2020.100816 32771971 PMC7412862

[B86] Samimi S. Maghsoudnia N. Eftekhari R. B. Dorkoosh F. (2019). Lipid-based nanoparticles for drug delivery systems. Charact. Biol. Nanomater. drug Deliv., 47–76. 10.1016/b978-0-12-814031-4.00003-9

[B87] Sanchez-Lievanos K. R. Tariq M. Brennessel W. W. Knowles K. E. (2020). Heterometallic trinuclear oxo-centered clusters as single-source precursors for synthesis of stoichiometric monodisperse transition metal ferrite nanocrystals. Dalton Trans. 49, 16348–16358. 10.1039/d0dt01369b 32432619

[B88] Sathiyaseelan A. Saravanakumar K. Mariadoss A. V. A. Wang M.-H. (2021). pH-controlled nucleolin targeted release of dual drug from chitosan-gold based aptamer functionalized nano drug delivery system for improved glioblastoma treatment. Carbohydr. Polym. 262, 117907. 10.1016/j.carbpol.2021.117907 33838795

[B89] Sheta S. M. El-Sheikh S. M. Abd-Elzaher M. M. (2018). Simple synthesis of novel copper metal–organic framework nanoparticles: biosensing and biological applications. Dalton Trans. 47, 4847–4855. 10.1039/c8dt00371h 29541717

[B90] Shi Z. Chen X. Zhang L. Ding S. Wang X. Lei Q. (2018). FA-PEG decorated MOF nanoparticles as a targeted drug delivery system for controlled release of an autophagy inhibitor. Biomaterials Sci. 6, 2582–2590. 10.1039/c8bm00625c 30151542

[B91] Song H. Su C. Cui W. Zhu B. Liu L. Chen Z. (2013). Folic acid-chitosan conjugated nanoparticles for improving tumor-targeted drug delivery. BioMed Res. Int. 2013, 723158. 10.1155/2013/723158 24282819 PMC3825055

[B92] Stella B. Arpicco S. Peracchia M. T. Desmaële D. Hoebeke J. Renoir M. (2000). Design of folic acid‐conjugated nanoparticles for drug targeting. J. Pharm. Sci. 89, 1452–1464. 10.1002/1520-6017(200011)89:11<1452::aid-jps8>3.0.co;2-p 11015690

[B93] Sun Y. Davis E. (2021). Nanoplatforms for targeted stimuli-responsive drug delivery: A review of platform materials and stimuli-responsive release and targeting mechanisms. Nanomaterials 11, 746. 10.3390/nano11030746 33809633 PMC8000772

[B94] Surekha B. Kommana N. S. Dubey S. K. Kumar A. P. Shukla R. Kesharwani P. (2021). PAMAM dendrimer as a talented multifunctional biomimetic nanocarrier for cancer diagnosis and therapy. Colloids Surfaces B Biointerfaces 204, 111837. 10.1016/j.colsurfb.2021.111837 33992888

[B95] Taghavi S. Ramezani M. Alibolandi M. Abnous K. Taghdisi S. M. (2017). Chitosan-modified PLGA nanoparticles tagged with 5TR1 aptamer for *in vivo* tumor-targeted drug delivery. Cancer Lett. 400, 1–8. 10.1016/j.canlet.2017.04.008 28412238

[B96] Tesauro D. Accardo A. Diaferia C. Milano V. Guillon J. Ronga L. (2019). Peptide-based drug-delivery systems in biotechnological applications: recent advances and perspectives. Molecules 24, 351. 10.3390/molecules24020351 30669445 PMC6359574

[B97] Torchilin V. P. (2010). Passive and active drug targeting: drug delivery to tumors as an example. Drug Deliv., 3–53. 10.1007/978-3-642-00477-3_1 20217525

[B98] Trushina D. B. Sapach A. Y. Burachevskaia O. A. Medvedev P. V. Khmelenin D. N. Borodina T. N. (2022). Doxorubicin-loaded core–shell UiO-66@ SiO2 metal–organic frameworks for targeted cellular uptake and cancer treatment. Pharmaceutics 14, 1325. 10.3390/pharmaceutics14071325 35890221 PMC9324125

[B99] Ulldemolins A. Seras-Franzoso J. Andrade F. Rafael D. Abasolo I. Gener P. (2021). Perspectives of nano-carrier drug delivery systems to overcome cancer drug resistance in the clinics. Cancer Drug Resist. 4, 44–68. 10.20517/cdr.2020.59 35582007 PMC9019183

[B100] Vahed S. Z. Salehi R. Davaran S. Sharifi S. (2017). Liposome-based drug co-delivery systems in cancer cells. Mater. Sci. Eng. C 71, 1327–1341. 10.1016/j.msec.2016.11.073 27987688

[B101] Valencia-Lazcano A. A. Hassan D. Pourmadadi M. Behzadmehr R. Rahdar A. Medina D. I. (2023). 5-Fluorouracil nano-delivery systems as a cutting-edge for cancer therapy. Eur. J. Med. Chem. 246, 114995. 10.1016/j.ejmech.2022.114995 36493619

[B102] van der Meel R. Sulheim E. Shi Y. Kiessling F. Mulder W. J. Lammers T. (2019). Smart cancer nanomedicine. Nat. Nanotechnol. 14, 1007–1017. 10.1038/s41565-019-0567-y 31695150 PMC7227032

[B103] Vodyashkin A. A. Sergorodceva A. V. Kezimana P. Stanishevskiy Y. M. (2023). Metal-organic framework (MOF)—a universal material for biomedicine. Int. J. Mol. Sci. 24, 7819. 10.3390/ijms24097819 37175523 PMC10178275

[B104] Wang Y. Yan J. Wen N. Xiong H. Cai S. He Q. (2020). Metal-organic frameworks for stimuli-responsive drug delivery. Biomaterials 230, 119619. 10.1016/j.biomaterials.2019.119619 31757529

[B105] Wang L. You X. Dai C. Fang Y. Wu J. (2022a). Development of poly (p-coumaric acid) as a self-anticancer nanocarrier for efficient and biosafe cancer therapy. Biomaterials Sci. 10, 2263–2274. 10.1039/d2bm00027j 35362499

[B106] Wang C. Xue F. Wang M. An L. Wu D. Tian Q. (2022b). 2D Cu-bipyridine MOF nanosheet as an agent for colon cancer therapy: A three-in-one approach for enhancing chemodynamic therapy. ACS Appl. Mater. Interfaces 14, 38604–38616. 10.1021/acsami.2c11999 35979620

[B107] Wang L. Xu Y. Liu C. Si W. Wang W. Zhang Y. (2022c). Copper-doped MOF-based nanocomposite for GSH depleted chemo/photothermal/chemodynamic combination therapy. Chem. Eng. J. 438, 135567. 10.1016/j.cej.2022.135567

[B108] Wen J. Liu X. Liu L. Ma X. Fakhri A. Gupta V. K. (2021). Bimetal cobalt-Iron based organic frameworks with coordinated sites as synergistic catalyst for fenton catalysis study and antibacterial efficiency. Colloids Surfaces A Physicochem. Eng. Aspects 610, 125683. 10.1016/j.colsurfa.2020.125683

[B109] Wongsakulphasatch S. Nouar F. Rodriguez J. Scott L. Le Guillouzer C. Devic T. (2015). Direct accessibility of mixed-metal (III/II) acid sites through the rational synthesis of porous metal carboxylates. Chem. Commun. 51, 10194–10197. 10.1039/c5cc02550h 26015999

[B110] Xiao K. Shu B. Lv K. Huang P. Chang Q. Wu L. (2023). Recent progress of MIL MOF materials in degradation of organic pollutants by fenton reaction. Catalysts 13, 734. 10.3390/catal13040734

[B111] Xie B.-X. Shu W. Wang H.-S. Chen L. Xu J. Zhang F.-Z. (2022). Folic acid-modified metal-organic framework carries CPT and DOX for cancer treatment. J. Solid State Chem. 306, 122803. 10.1016/j.jssc.2021.122803

[B112] Yan J. Liu C. Wu Q. Zhou J. Xu X. Zhang L. (2020). Mineralization of pH-sensitive doxorubicin prodrug in ZIF-8 to enable targeted delivery to solid tumors. Anal. Chem. 92, 11453–11461. 10.1021/acs.analchem.0c02599 32664723 PMC7458362

[B113] Yang X. Zhang C. Deng D. Gu Y. Wang H. Zhong Q. (2022). Multiple stimuli‐responsive MXene‐based hydrogel as intelligent drug delivery carriers for deep chronic wound healing. Small 18, 2104368. 10.1002/smll.202104368 34821453

[B114] Ye G. Wan L. Zhang Q. Liu H. Zhou J. Wu L. (2023). Boosting catalytic performance of MOF-808 (Zr) by direct generation of rich defective Zr nodes via a solvent-free approach. Inorg. Chem. 62, 4248–4259. 10.1021/acs.inorgchem.2c04364 36857420

[B115] Yin X. Alsuwaidi A. Zhang X. (2022). Hierarchical metal-organic framework (MOF) pore engineering. Microporous Mesoporous Mater. 330, 111633. 10.1016/j.micromeso.2021.111633

[B116] Yu C. Zhang Y. Wang N. Wei W. Cao K. Zhang Q. (2022). Treatment of bladder cancer by geoinspired synthetic chrysotile nanocarrier-delivered circPRMT5 siRNA. Biomaterials Res. 26, 6–20. 10.1186/s40824-022-00251-z PMC8818206 35123588

[B117] Yuan C.-Z. Jiang Y.-F. Wang Z. Xie X. Yang Z.-K. Yousaf A. B. (2016). Cobalt phosphate nanoparticles decorated with nitrogen-doped carbon layers as highly active and stable electrocatalysts for the oxygen evolution reaction. J. Mater. Chem. A 4, 8155–8160. 10.1039/c6ta01929c

[B118] Zeng X. Chen B. Song Y. Lin X. Zhou S.-F. Zhan G. (2021). Fabrication of versatile hollow metal–organic framework nanoplatforms for folate-targeted and combined cancer imaging and therapy. ACS Appl. Bio Mater. 4, 6417–6429. 10.1021/acsabm.1c00603 35006919

[B119] Zeng H. Xia C. Zhao B. Zhu M. Zhang H. Zhang D. (2022). Folic acid–functionalized metal-organic framework nanoparticles as drug carriers improved bufalin antitumor activity against breast cancer. Front. Pharmacol. 12, 747992. 10.3389/fphar.2021.747992 35115921 PMC8805731

[B120] Zhang T.-z. Lu Y. Li Y.-g. Zhang Z. Chen W.-l. Fu H. (2012). Metal–organic frameworks constructed from three kinds of new Fe-containing secondary building units. Inorganica Chim. Acta 384, 219–224. 10.1016/j.ica.2011.12.006

[B121] Zhang H.-J. Zhao X. Chen L.-J. Yang C.-X. Yan X.-P. (2020). Dendrimer grafted persistent luminescent nanoplatform for aptamer guided tumor imaging and acid-responsive drug delivery. Talanta 219, 121209. 10.1016/j.talanta.2020.121209 32887113

[B122] Zhao H. Zhao Y. Liu D. (2021). pH and H2S dual-responsive magnetic metal–organic frameworks for controlling the release of 5-fluorouracil. ACS Appl. Bio Mater. 4, 7103–7110. 10.1021/acsabm.1c00710 35006942

[B123] Zhao X. He S. Li B. Liu B. Shi Y. Cong W. (2023). DUCNP@ Mn–MOF/FOE as a highly selective and bioavailable drug delivery system for synergistic combination cancer therapy. Nano Lett. 23, 863–871. 10.1021/acs.nanolett.2c04042 36651872

