# Supplementary material for: A pH-responsive bi-MIL-88B MOF coated with folic acid-conjugated chitosan as a promising nanocarrier for targeted drug delivery of 5-Fluorouracil
Source: Front Pharmacol. 2023 Sep 6;14:1265440. doi: 10.3389/fphar.2023.1265440 (PMC10517339; doi:10.3389/fphar.2023.1265440)
Supplement: Supplementary file 1 [file DataSheet1.docx]

A pH-responsive bi-MIL-88B MOF coated with folic acid-conjugated chitosan as a promising nanocarrier for targeted drug delivery of 5-Fluorouracil

Muhammad Usman Akbar ^1^, Saadullah Khattak ^2^, Malik Ihsanullah Khan ^3^, Umair Ali Khan Saddozai ^4^, Nemat Ali ^5^, Abdullah F. AlAsmari ^5^, Muhammad Zaheer ^6^* and Muhammad Badar ^1^*

^1.^ Gomal Center of Biochemistry and Biotechnology, Gomal University, Dera Ismail Khan, 29050, Pakistan; usman.akber39@gmail.com, mbadar@gu.edu.pk

^2.^  Henan International Joint Laboratory of Nuclear Protein Regulation, School of Basic Medical Sciences, Henan University, Kaifeng 475004, China [saadullah@henu.edu.cn](mailto:saadullah@henu.edu.cn)

^3.^ Institute of Molecular Biology and Biotechnology, The University of Lahore, Lahore, 54000, Pakistan; ihsan.ullah@imbb.uol.edu.pk

^4.^ Department of Preventive Medicine, Institute of Bioinformatics, Henan Provincial Engineering Center for Tumor Molecular Medicine, School of Basic Medical Sciences, Henan University, Kaifeng 475004, China; umairsaddozai2@gmail.com

^5..^ Department of Pharmacology and Toxicology, College of Pharmacy, King Saud University, Riyadh 11451, Saudi Arabia nali1@ksu.edu.sa, afalasmari@ksu.edu.sa

^6.^ Department of Chemistry and Chemical Engineering, Syed Babar Ali School of Science and Engineering, Lahore University of Management Sciences (LUMS), Lahore, 54792, Pakistan; muhammad.zaheer@lums.edu.pk

***** Correspondence: [muhammad.zaheer@lums.edu.pk](mailto:mmubarak@ju.edu.jo) (M.Z.); [mbadar@gu.edu.pk](mailto:mbadar@gu.edu.pk) (M.B.)

**Table of Contents**

**Figure S1.** (a-b) SEM images; (c) PXRD pattern and (d) Elemental maps of FeCo cluster. 2

**Figure S2.** EDX spectra of (a) FeCo Cluster and (b) bi-MIL-88B MOFs. 2

**Figure S3.** FT-IR spectra of FeCo cluster and bi-MIL-88B MOFs. 3

**Figure S4.** (a) N_2_ adsorption-desorption isotherm and (b) pore dynamics of bi-MIL-88B MOFs. 3

**Figure S5.** FT-IR spectra of bi-MIL-88B, 5-FU and 5-FU@bi-MIL-88B MOFs. 4

**Figure S6.** FT-IR spectra of FA, CS and FC. 4

**Figure S7**. 5-FU calibration curve in ethanol. 5

**Figure S8.** 5-FU calibration curve in PBS. 5

**Table S1**. Elemental composition of FeCo cluster and bi-MIL-88B MOFs evaluated by EDX and ICP-OES……. 3


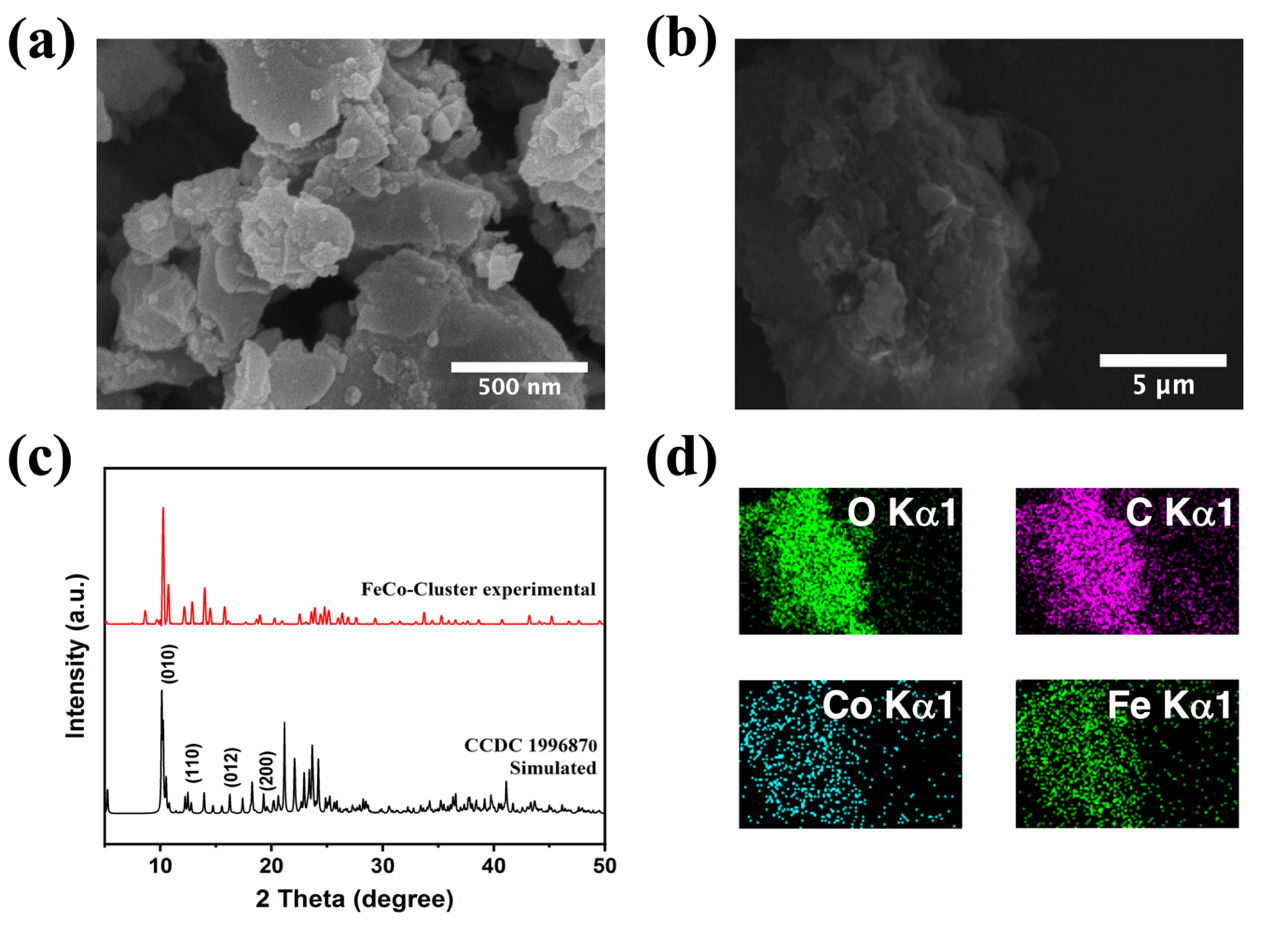


**Figure S1.** (a-b) SEM images; (c) PXRD pattern and (d) Elemental maps of FeCo cluster.


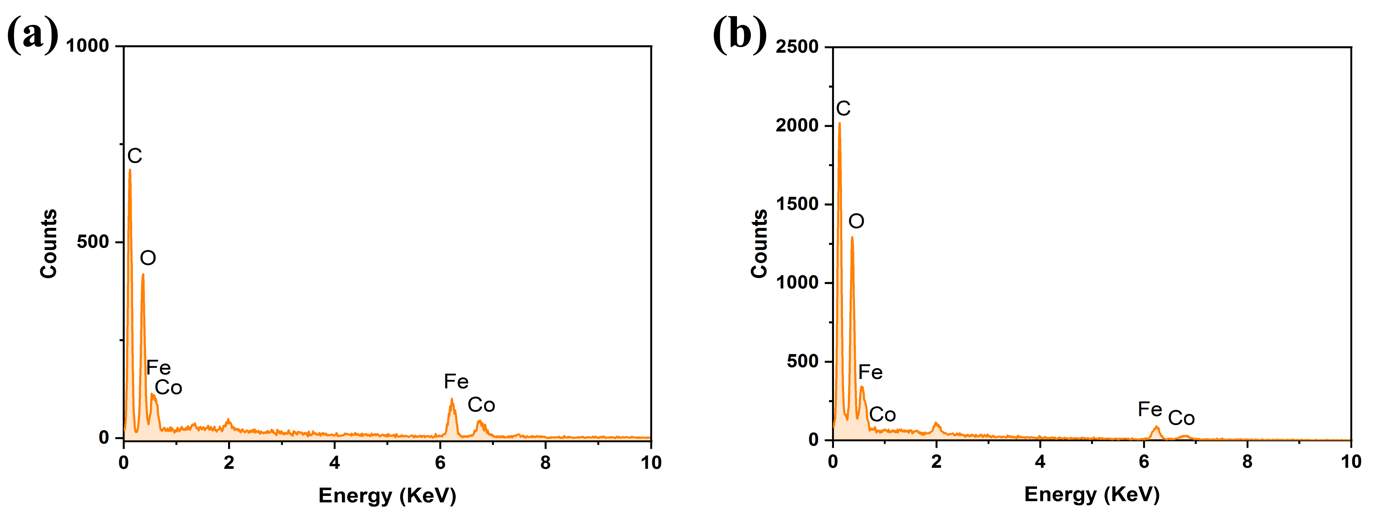


**Figure S2.** EDX spectra of (a) FeCo Cluster and (b) bi-MIL-88B MOFs.

**Table S1**. Elemental composition of FeCo cluster and bi-MIL-88B MOFs evaluated by EDX and ICP-OES.

| **Sample** | **EDX**  n(Fe) : n(Co) | **ICP-OES**  n(Fe) : n(Co) |
| --- | --- | --- |
| FeCo Cluster | 2.01 : 1 | 1.96 : 1 |
| bi-MIL-88B | 1.96 : 1 | 1.94 : 1 |


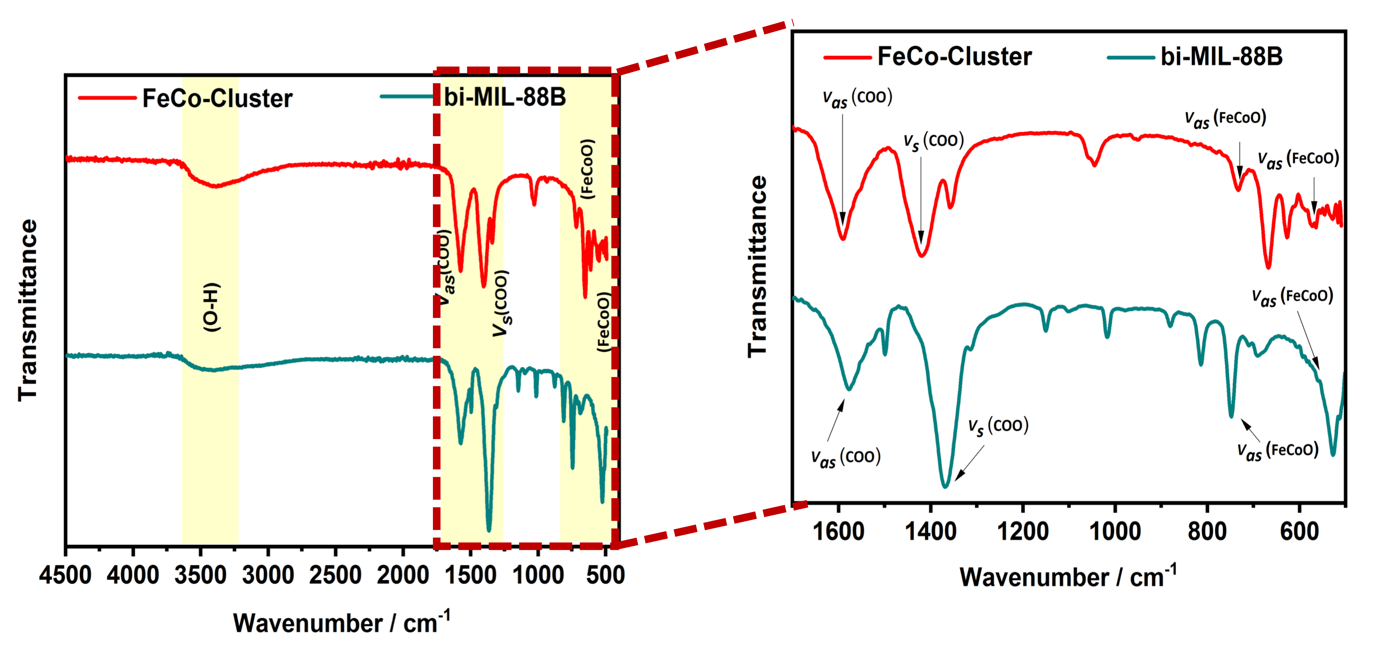


**Figure S3.** FT-IR spectra of FeCo cluster and bi-MIL-88B MOFs.


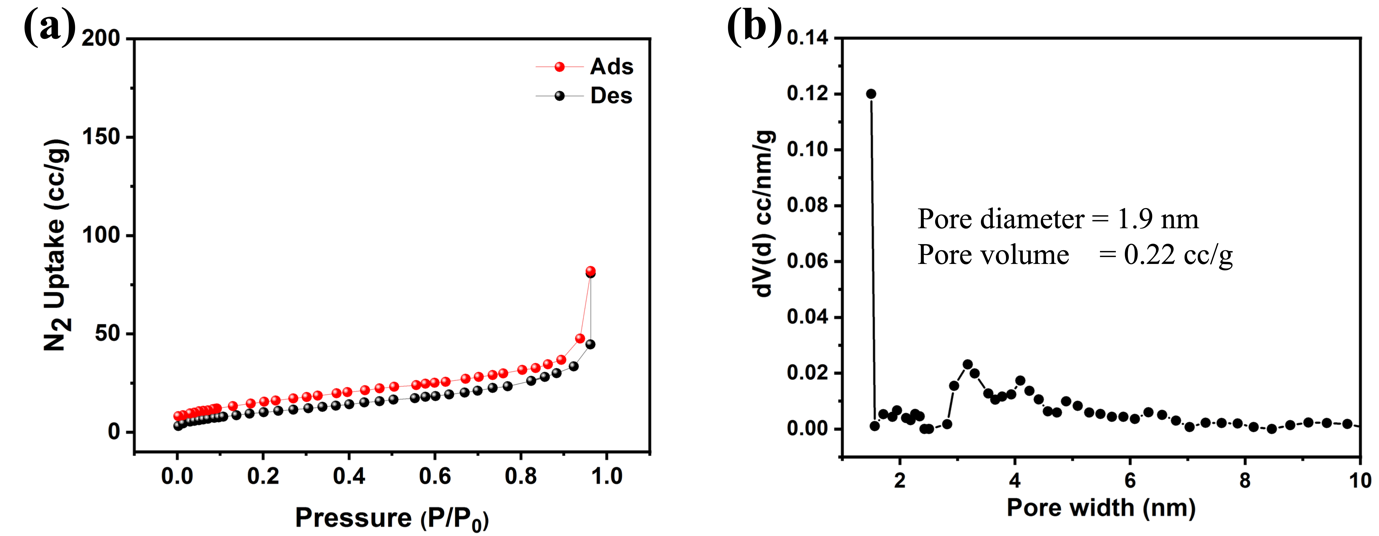


**Figure S4.** (a) N_2_ adsorption-desorption isotherm and (b) pore dynamics of bi-MIL-88B MOFs.


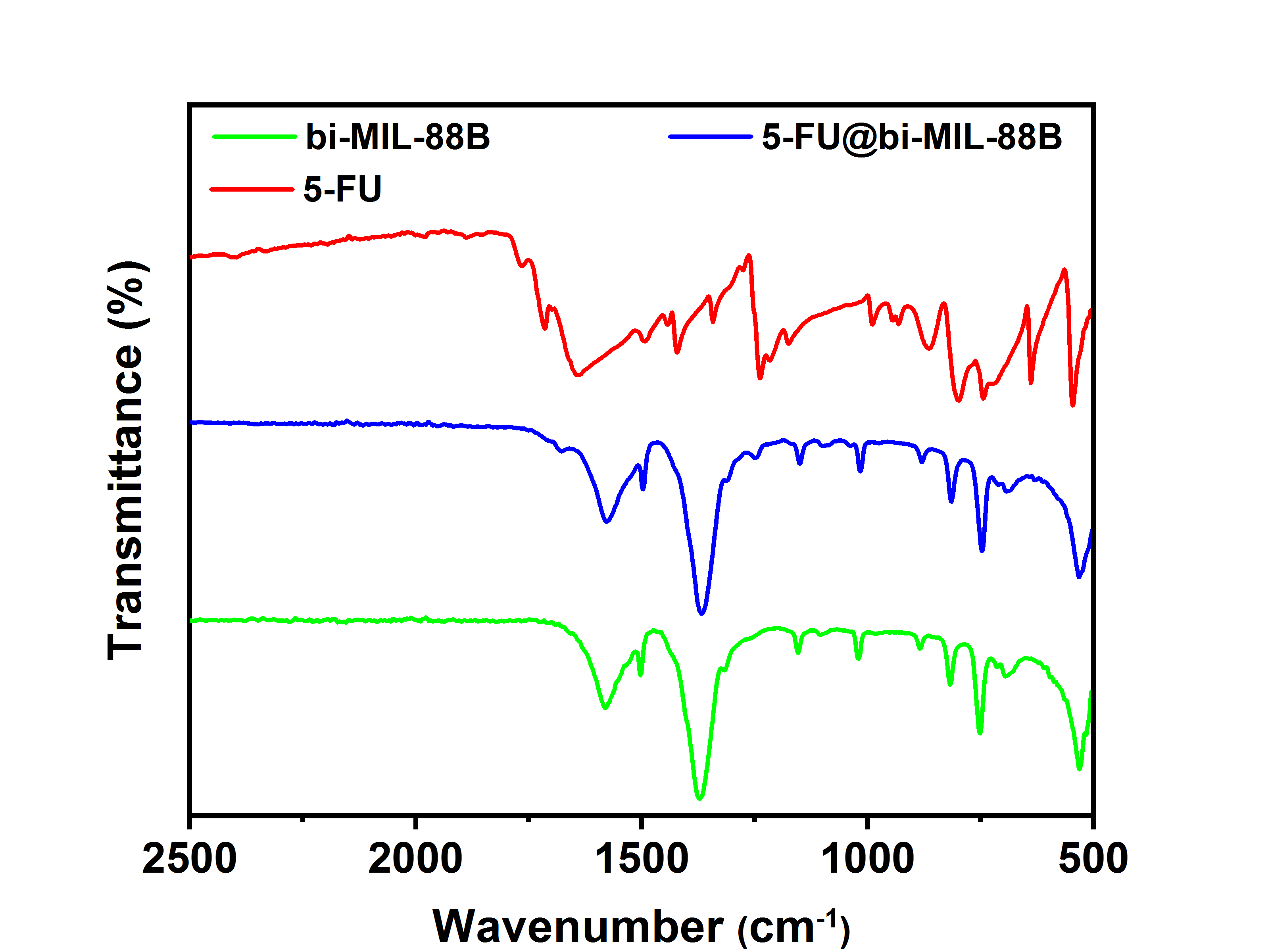


**Figure S5.** FT-IR spectra of bi-MIL-88B, 5-FU and 5-FU@bi-MIL-88B MOFs.


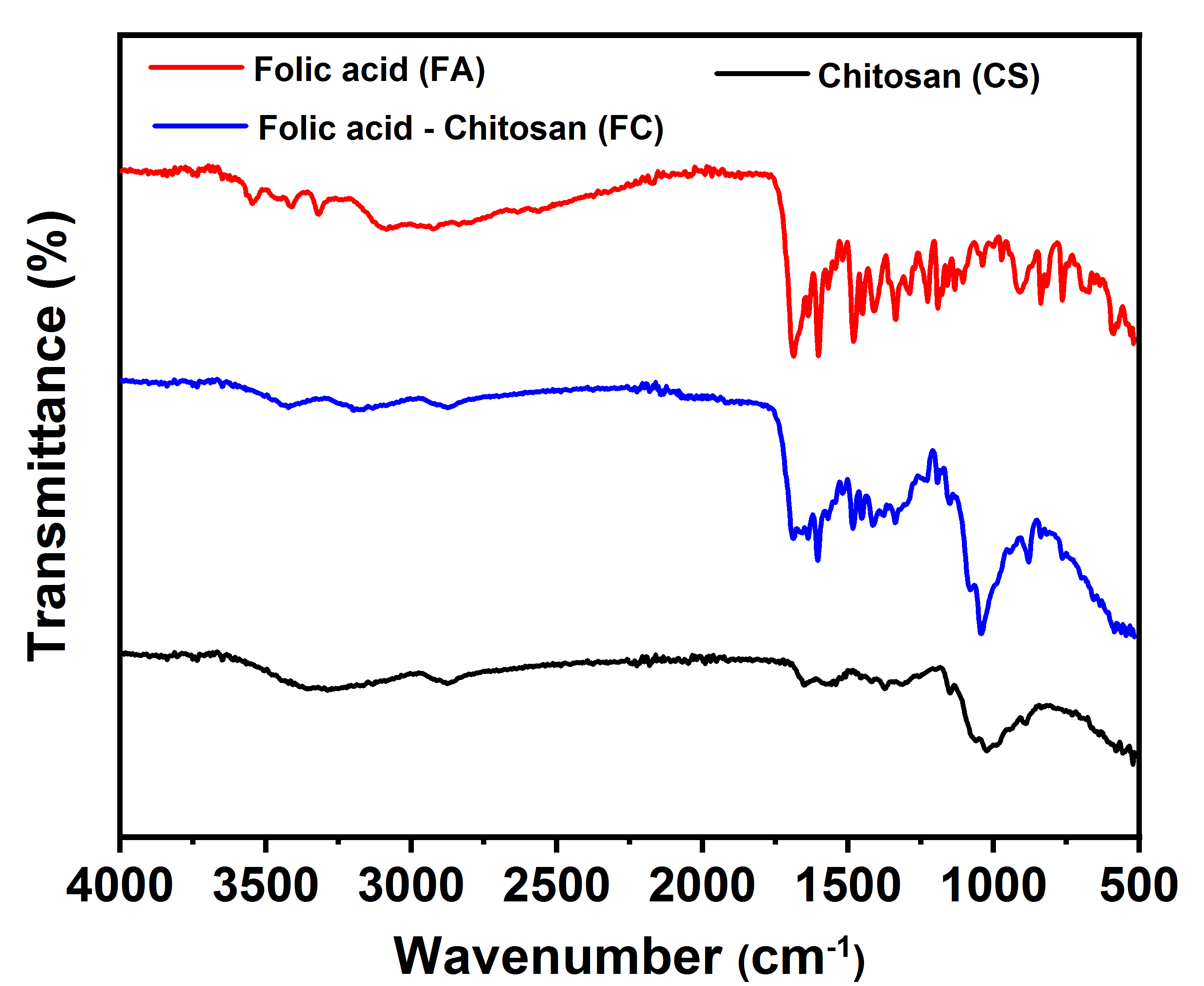


**Figure S6.** FT-IR spectra of FA, CS and FC.


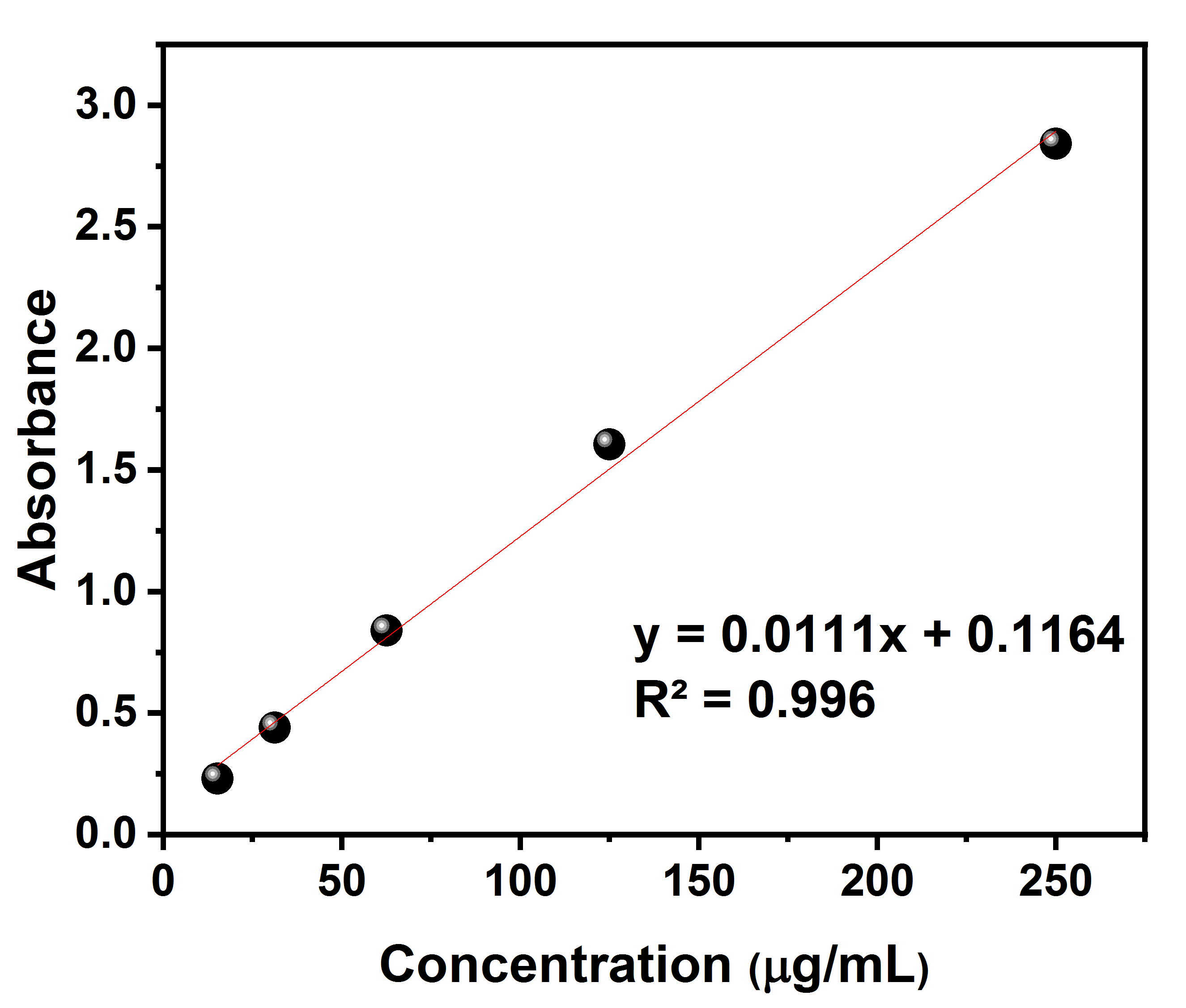


**Figure S7**. 5-FU calibration curve in ethanol.


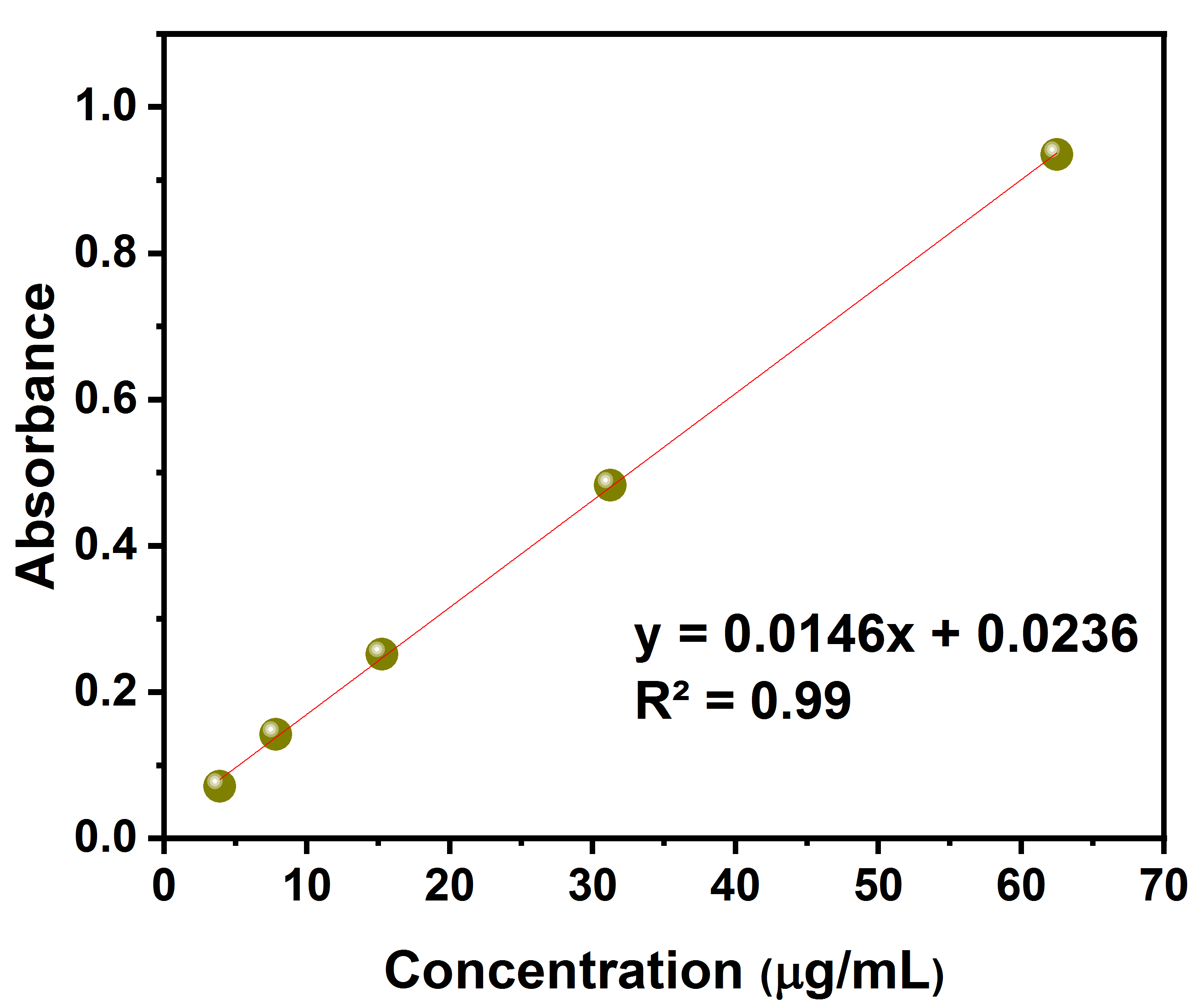


**Figure S8.** 5-FU calibration curve in PBS.
